# Prevalence and severity of anxiety and depression in Chinese patients with breast cancer: a systematic review and meta-analysis

**DOI:** 10.3389/fpsyt.2023.1080413

**Published:** 2023-06-28

**Authors:** Fuying Tao, Mengnan Xu, Qi Zou, Lin Tang, Jianping Feng, Zhenyu Li

**Affiliations:** ^1^Department of Anesthesiology and Perioperative Medicine, The First Affiliated Hospital of Nanjing Medical University, Nanjing, Jiangsu, China; ^2^School of Nursing, Nanjing Medical University, Nanjing, Jiangsu, China; ^3^Department of Nursing, The First Affiliated Hospital of Nanjing Medical University, Nanjing, Jiangsu, China

**Keywords:** anxiety, depression, breast cancer, China, meta-analysis

## Abstract

**Context:**

Anxiety/depression in breast cancer (BC) is common around the world, and Chinese BC patients should not be ignored. The prevalence of anxiety and depression among BC patients are various in different regions of China, but no clear summarization has been made.

**Purpose:**

This meta-analysis aimed to evaluate the prevalence and severity of anxiety and depression among breast cancer (BC) patients in China.

**Methods:**

A literature search on PubMed, Web of Science, Embase, CINAHL, Scopus, PsycINFO, Cochrane database library, CNKI, Wanfang, and SinoMed was conducted up to 29 December 2021. The effect size (ES) or standard mean difference (SMD) and the corresponding 95% confidence intervals (CIs) for the prevalence and severity of anxiety/depression were calculated using the STATA 12.0 software.

**Results:**

A total of 63 identified studies were included, containing a total of 53,513 Chinese women confirmed breast cancer. The results showed a high pooled prevalence of anxiety (38%, 95% CI, 27–50%, *I*^2^ = 99.4%, *p* < 0.001) and depression (38%, 95% CI, 33–44%, *I*^2^ = 99.2%, *p* < 0.001) among Chinese BC patients. Moreover, both anxiety (SMD = 0.30, 95% CI, 0.08–0.53, *I*^2^ = 91.6%, *p* < 0.001) and depression (SMD = 0.25, 95% CI, −0.05–0.55, *I*^2^ = 95.3%, *p* < 0.001) in BC patients were more serious than those in healthy controls, but not significantly different from patients with other diseases. Specifically, among the six regions included, the prevalence of anxiety and depression were both the highest in Northeast China, obviously superior than the second-highest region.

**Conclusion:**

The study showed high levels of anxiety and depression among BC patients in China, especially those in the northeast. Clinicians and researchers should pay attention to the psychological problems of patients with breast cancer and regard it as one of the important prognostic outcomes of patients.

**Systematic review registration:**

https://www.crd.york.ac.uk/prospero/index.php, PROSPERO: CRD42020151752.

## Introduction

Breast cancer (BC) is a malignant tumor that arises from the epithelial cells in the ducts or lobules of the breast gland (1). According to 2020 global cancer statistics, BC surpassed lung cancer to become the most frequently diagnosed cancer for the first time (2). With the improvement in survival rates and life expectancy among BC patients, mental health has gradually gained recognition as an important indicator for assessing long-term prognosis (3–5). It is worth noting that anxiety and depression are prevalent psychological symptoms experienced by BC patients during the recovery phase (6–9).

BC patients with anxiety and/or depression often experience more severe physical and mental health problems, including disease deterioration, cancer recurrence, body image disturbance, impaired relationships, sleep disturbances, suicidal ideation, and suicidal behavior, which ultimately contribute to increased patient mortality (8, 10–13). Previous studies have consistently reported a rising prevalence of anxiety and depression among women with BC worldwide (6, 14). An investigative study conducted in Iran found that the prevalence of anxiety and depression was 60 and 66.6%, respectively (15). Another study conducted in British Columbia showed the rates of anxiety and depression were 58.6 and 33.6%, respectively (16). In addition, Liu et al. (6) reported that, in Northeast China, over 70% of BC patients suffered from anxiety or depressive symptoms. A survey conducted in Shanghai also revealed that the prevalence of major depression among Chinese BC survivors was 20.59% and the risk for depression within the 1st year was double than in more than 1 year after surgery (17).

It can be seen, anxiety/depression in BC is common around the world, and Chinese BC patients should not be ignored. Several studies have shown notable variations in the prevalence of anxiety and depression among BC patients across different regions of China. However, no comprehensive quantitative review specifically focusing on anxiety and depression among Chinese BC patients has been conducted. While Pilevarzadeh et al. performed reviews on anxiety and depression of BC patients worldwide, Chinese BC patients were not analyzed separately (18, 19). Although another meta-analysis investigated the depression of BC patients in Iran (20), its finding cannot be easily generalized to Chinese BC patients due to different backgrounds (21). Considering the unique cultural, economic, and healthcare contexts, the prevalence and severity of anxiety and depression in Chinese BC patients are different from those in other countries. Consequently, examining anxiety and depression will help to increase public awareness and provide a reference for health care policies. However, even if reliable estimates of anxiety and depression are important for the prevention and treatment of Chinese BC patients, they have not yet been clearly summarized.

To address this limitation, we conducted this systematic review and meta-analysis with the following aims: (1) establish the pooled prevalence and severity of anxiety/depression in Chinese adult BC patients; (2) summarize the methods used to define anxiety and depression in BC. Through this meta-analysis, healthcare providers could gain a comprehensive understanding of anxiety and depression among Chinese BC patients, enabling them to develop more precise treatment strategies for enhancing their mental health.

## Methods

We conducted this systematic review within the Reporting Items for Meta-analysis of Observational Studies in Epidemiology (MOOSE) statement (22) and following a predetermined registered protocol PROSPERO: CRD42020151752, https://www.crd.york.ac.uk/prospero/index.php.

### Search strategy

A systematic review of the literature published in scientific journals from their inception until 29 December 2021 was conducted by two independent researchers. The review included seven English and three Chinese databases: PubMed, Web of Science, Embase, CINAHL, Scopus, PsycINFO, Cochrane database library, CNKI (China Knowledge Resource Integrated Database), Wanfang, and SinoMed (Chinese Biomedicine Literature Database). The search strategy involved the use of keywords such as “anxiety disorder,” “Anxiety,” “Depressive Disorder,” “depression,” “Dysthymic Disorder,” “Mood Disorders,” “Breast cancer,” “Breast neoplasm,” and “China/Chinese.” The retrieval steps for PubMed are shown in Supplementary material 1. Additionally, a citation chasing search strategy was conducted with all reference and relevant reviews to identify potentially omitted articles.

### Inclusion and exclusion criteria

The following inclusion criteria were used: (1) Chinese patients with BC; (2) cross-sectional, cohort or case-control studies; (3) studies reporting the prevalence or mean with standard deviation (SD); (4) validated methods, such as clinical interviews or self-report instruments, used to assess anxiety or depression; (5) studied involving adults aged at least 18 years old; (6) Chinese literature published in core journal; and (7) studies with a sample size of no <5 (23). The following exclusion criteria were applied: (1) studies published in languages other than Chinese or English; (2) duplicated and useless data.

### Data extraction

The titles and abstracts for eligibility was screened by two researchers (TF and XM) independently. Then, they reviewed the full text in the remaining studies to confirm that eligibility criteria were met. Conflicts were resolved with ZQ through discussion. Data were extracted by one trained investigator (TL) and checked by two others (TF and XM). Abstracted items included year, author, region, sample size, the average age of participants, percentage of marriage or cohabitation, study design, criteria for detection of anxiety or depression, reported prevalence or scale score of anxiety or depression, and type of control group. When multiple studies reported data for the same outcome and sample, we included one study on the basis of largest sample size or data presentation (closest fit to study requirements). For studies with missing data, we attempted to contact the authors for additional information and used appropriate imputation techniques when necessary. In studies that only presented the mean with SD of ages for BC and control group, we used the formula to calculate the overall mean with SD.

### Quality assessment

We used Hoy's critical appraisal checklist for quality evaluation of each study included in the present meta-analysis (24). This 10-item checklist has two dimensions: external validity (target population, minimal non-response bias, sampling frame, and method are assessed by items 1–4) and internal validity (case definition, study instrument, the method, and mode of data collection are assessed by items 5–9, and item 10 assesses bias related to the analysis). The finally assessment evaluates the overall risk of study bias, judging to be at low, moderate, or high risk of bias. Each study was evaluated by two independent researchers (TL and ZQ) while disagreements resolved through third reviewers (TF).

### Outcome measures

The outcomes were mean with SD or prevalence of anxiety and depression diagnosed with a structured clinical assessment [e.g., Diagnostic and Statistical Manual of Mental Disorders (DSM)-IV or International Classification of Diseases (ICD)-9] or validated assessment tools [e.g., the Hospital Anxiety and Depression Scale (HADS), Generalized Anxiety Disorder Questionnaire (GAD-7), Patient Health Questionnaire (PHQ-9), and the Center for Epidemiologic Studies Depression Scale (CES-D)]. The primary outcome of this meta-analysis were the pooled prevalence and severity of anxiety and depression in Chinese BC patients, while secondary outcomes included the comparison of anxiety and depression levels across different regions, control group types, and diagnostic criteria.

### Statistical analyses

For categorical variables, we calculated the effect size (ES) with 95% confidence intervals (CI). For numerical variables, standard mean difference (SMD) with CI was calculated. We chose the random-effects model because it accounts for potential between-study heterogeneity and provides more conservative estimates when heterogeneity is present (25). In addition, the forest plots were used to visually present the results of overall and within subgroups. We used *I*^2^ to assess the between-study heterogeneity with thresholds of ≥25, ≥50, and ≥75% representing low, moderate, and high heterogeneity, respectively (26). The sensitivity analyses were conducted to explore the effect of a single study on the overall prevalence estimate by serially excluding each study. The heterogeneity on region, the type of control group, criteria for detection of anxiety or depression were determined in subgroup analysis if there were at least two studies in the subgroup. Egger's test alliance with funnel plots was used to explore the potential publication bias (27, 28). Statistical analyses were performed with STATA 12.0 (StataCorp, Texas, USA, RRID: SCR_012763) statistical software. A two-sided test and a significant level of 0.05 were used.

## Results

### Research results

**Figure 1:**
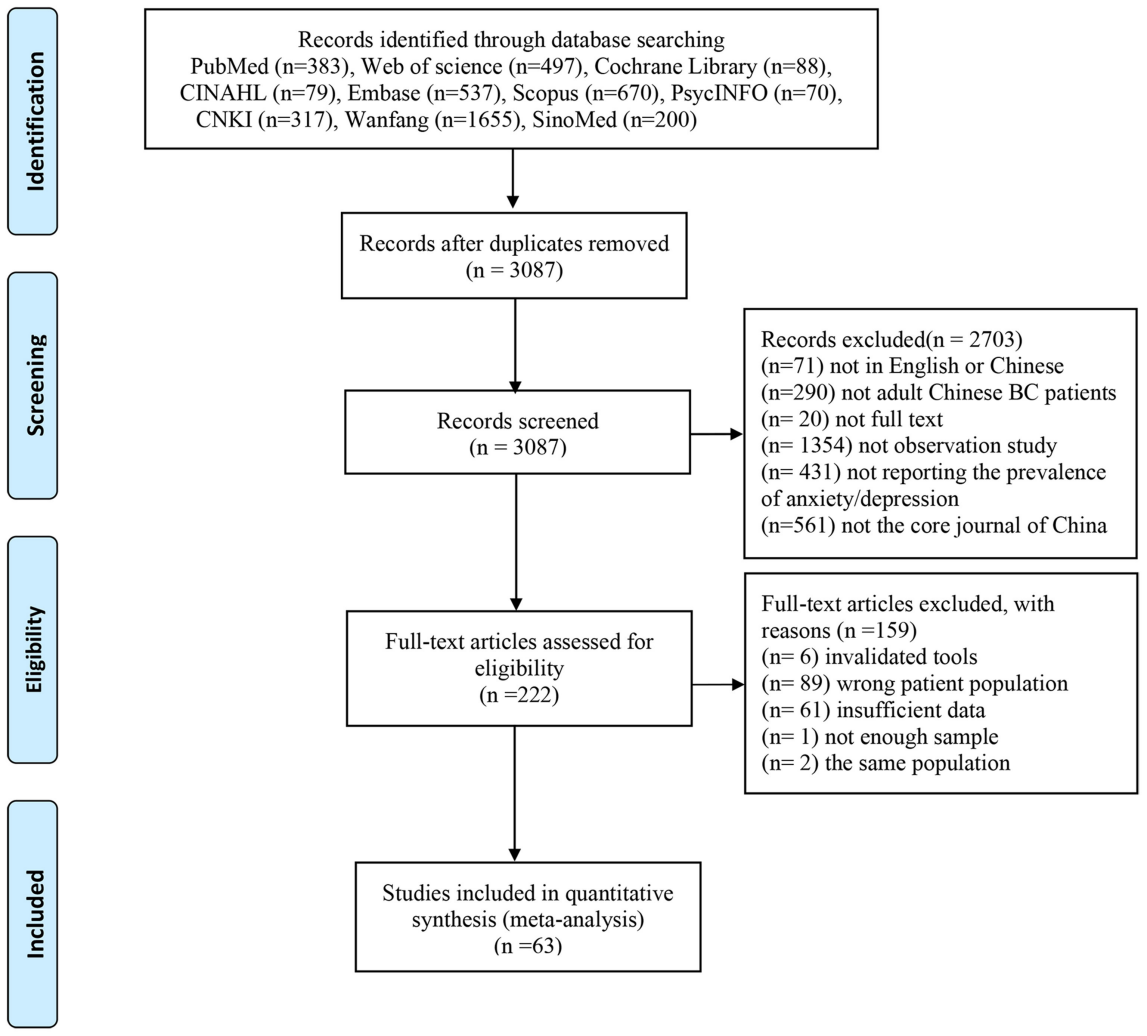
PRISMA flow diagram.

Figure 1 presents the details of the study selection process. The initial search identified a total of 4,496 articles through electronic databases. Among these, 1,409 duplicates were removed by using EndNote software. Then, after screening titles and abstracts of the remaining articles, 2,703 studies were excluded. Therefore, 222 were reviewed for further full-text, of which 63 articles were included finally.

### Study characteristics

The characteristics of the included studies were shown in Table 1. The complete references of the included studies can be seen in Supplementary material 2. A total of 63 identified studies proceeded to the data extraction stage, containing a total of 53,513 women with histologically confirmed BC. Of the 63 studies, the majority (*n* = 45 studies) were of cross-sectional design, whereas the remaining were case-control (*n* = 5), or cohort studies (*n* = 13). The location of studies spanned nationally and was prominent in East China (including Zhejiang, Shandong, Anhui, Jiangsu, Shanghai, Fujian, and Taiwan; *n* = 24), followed by South China (including Hong Kong and Guangdong; *n* = 13), Central China (including Henan, Hunan, and Hubei; *n* = 10), North China (including Beijing; *n* = 6), Northeast (including Heilongjiang and Liaoning; *n* = 4), and Southwest (including Sichuan; *n* = 2). The types of control groups include healthy controls (*n* = 9) and patients with other diseases controls (*n* = 6). When evaluated by Holy quality assessment criteria, 47 studies received low risk of bias, 16 received moderate risk of bias. The details of the quality assessment for 63 included studies were presented in Supplementary material 3.

**Table 1:** The main characteristics of the studies included in the present systematic review and meta-analysis.

**References**	**Region**	**Sample size**	**Age, mean ±SD/median, years**	**Design**	**Anxiety, prevalence (%)/score (M ±SD)**	**Depression, prevalence (%)/score (M ±SD)**	**Control group**	**Risk of bias**
Zhao (2021)	Central China	151	37.38 ± 4.96	Cohort	-	56.29	-	Low
Zhu et al. (30)	East China	111	-	Cross-sectional	89.91	-	-	Moderate
Zhou (2020)	North China	109	42 ± 6	Cross-sectional	20.2	15.7	-	Low
Wen (2020)	Southwest	316	48.8 ± 0.52	Cross-sectional	12.3	19.0	-	Moderate
Chen (2020)	Multiple	625	49.12 ± 8.39	Cross-sectional	22.72	20.16	-	Low
Gao (2019)	North China	65	50 ± 8	Cross-sectional	16.92	-	-	Low
Lv (2017)	East China	180	43.11 ± 8.24	Cross-sectional	-	62.22	-	Low
Liu (2017)	Southwest	95	-	Cross-sectional	4.48 ± 0.20	6.94 ± 3.41	Health controls	Low
Xu (2016)	East China	174	53.86 ± 12.34	Cross-sectional	29.31	-	-	Low
Yang (2015)	East China	63	48.87 ± 50.53	Cross-sectional	-	46.03	-	Low
Zhang (2015)	South China	452	47.1 ± 9.8	Cross-sectional	61.6	50.6	-	Low
Xu (2015)	South China	205	46.4 ± 8.62	Cross-sectional	-	23.4	-	Moderate
Li (2015)	Central China	434	47.54 ± 7.72	Cohort	-	63.4	-	Low
Xu (2013)	East China	315	-	Cross-sectional	54.60	47.30	-	Moderate
Pang (2013)	North China	255	42.9 ± 17.7	Cross-sectional	14.9	11.6	-	Low
Zhou (2012)	East China	42	47.5 ± 8.53	Cross-sectional	-	33.33	-	Low
Zhang (2009)	Northwest	163	49.66 ± 9.22	Cross-sectional	36.1	43.5	-	Moderate
Liu (2008)	East China	102	38.31 ± 11.25	Cross-sectional	-	51.9	-	Moderate
Cheng (2016)	South China	30	51.67	Cross-sectional	4.63 ± 2.87	3.03 ± 2.67	Musculokeletal injury/healthy controls	Moderate
Ho (2013)	South China	181	48.3 ± 7.5	Cross-sectional	39.3	27.9	-	Low
Ho (2013)	South China	133	49 ± 8.2	Cross-sectional	32	31	-	Low
Lam (2018)	South China	140	55.81 ± 8.10	Cohort	22	21	-	Low
Li (2020)	Central China	658	46.98	Cross-sectional	56.4	47.3	-	Low
Li (2020)	East China	34	45.53 ± 10.09	Cross-sectional	-	4.20 ± 4.27	Depressive disorders/healthy controls	Low
Li (2016)	Central China	618	45.56 ± 6.41	Cross-sectional	32.1	32.1	-	Low
Li (2012)	South China	97	52.01 ± 10.15	Cross-sectional	3.1 ± 2.71	3.4 ± 3.40	Colorectal cancer	Low
Pan (2013)	North China	291	55.1 ± 6.40	Case control	1.38 ± 0.50	1.49 ± 0.54	Healthy controls	Moderate
Shih (2020)	East China	349	56.38 ± 10.12	Cross-sectional	-	7.2	-	Moderate
So (2009)	South China	215	51.65 ± 10.36	Cross-sectional	21	36	-	Low
So (2010)	South China	218	51.7 ± 10.32	Cross-sectional	21.1	34.4	-	Low
Sun (2019)	Central China	180	53.8 ± 15.2	Cross-sectional	21.1	16.7	-	Low
Tong (2017)	East China	57	-	Cross-sectional	-	59.6	-	Low
Zhao (2014)	North China	70	-	Cross-sectional	-	32.9	-	Low
Chen (2010)	East China	1,399	53.7 ± 9.8	Cohort	-	12.58	-	Low
Pan (2016)	East China	36,377	50.20 ± 11.70	Cohort	-	24.98	-	Low
Zhang (2017)	Northwest	410	41.67 ± 8.49	Cohort	64.15	65.37	-	Low
Li (2015)	Central China	247	47.45 ± 7.43	Cross-sectional	13.58 ± 7.15	72.4	Healthy controls	Low
Milbury (2017)	East China	97	46.4 ± 8.4	Cross-sectional	-	39.2	-	Low
Li (2011)	East China	252	46.00 ± 8.76	Cross-sectional	57.94	50.4	-	Low
Li et al. (5)	East China	120	40.85 ± 6.50	Case control	38.3	49.2	-	Low
Li (2017)	East China	131	44.87 ± 8.07	Cross-sectional	35.1	26.0	-	Low
Ho (2006)	South China	40	48.38 ± 5.99	Cross-sectional	37.73 ± 13.51	13.78 ± 11.49	Nasopharyngeal carcinoma	Low
Alagaratnam (1986)	-	23	45.2 ± 11.4	Cross-sectional	-	47.83	Other cancers	Moderate
Cui (2020)	Central China	207	48.59	Cross-sectional	62.8	51.2	Healthy controls	Low
Guo (2017)	Northwest	176	49.5	Cohort		59	-	Low
Chen (2021)	Central China	834	50.23	Cross-sectional	15.5	21.6	-	Low
Chen (2009)	East China	1,400	55.2 ± 9.8	Cohort	-	12.57	-	Low
Cheng (2018)	Multiple	267	38.45	Cross-sectional	5.43 ± 3.51	6.71 ± 3.56	Healthy controls	Low
Hong (2014)	East China	76	-	Cross-sectional	1.32	57.90	-	Low
Lan (2020)	North China	114	32.5	Cross-sectional	8.8	23.7	-	Low
Liu et al. (6)	Northwest	389	48.40	Cross-sectional	92.03	89.72	-	Low
Ng (2020)	South China	140	55.81 ± 8.10	Cross-sectional	22.14	4.43 ± 3.86	Healthy controls	Moderate
Qiu et al. (17)	East China	505	52.02 ± 4.55	Cohort	-	20.59	-	Low
Wang (2014)	Multiple	111	49.7 ± 9.6	Cross-sectional	42.34 ± 10.38	16.64 ± 11.29	Healthy controls	Low
Wang (2014)	Central China	1,013	48.31 ± 8.68	Cohort	-	60.33	-	Low
Wei (2019)	Central China	575	42.72 ± 11.99	Cross-sectional	-	27.0	-	Low
Wu (2020)	East China	128	50.57 ± 9.43	Cohort	53.125	28.125	-	Moderate
Zhang (2021)	South China	71	52.14 ± 9.19	Cross-sectional	36.6	33.8	-	Moderate
Zhang (2015)	East China	264	44.7 ± 7.2	Cohort	4.9	35.6	-	Low
Fielding (2014)	South China	218	56.7 ± 9.1	Cohort	5.5	6.4	-	Low
Huang (2019)	East China	63	48.87 ± 8.30	Case control	-	46.03	-	Moderate
Liu (2016)	East China	582	34.51 ± 4.61	Case control	-	45.7	-	Moderate
Wang (2019)	East China	156	47.5 ± 11.8	Case control	30.77	-	-	Moderate

### Prevalence and severity of anxiety among Chinese BC patients

**Figure 2:**
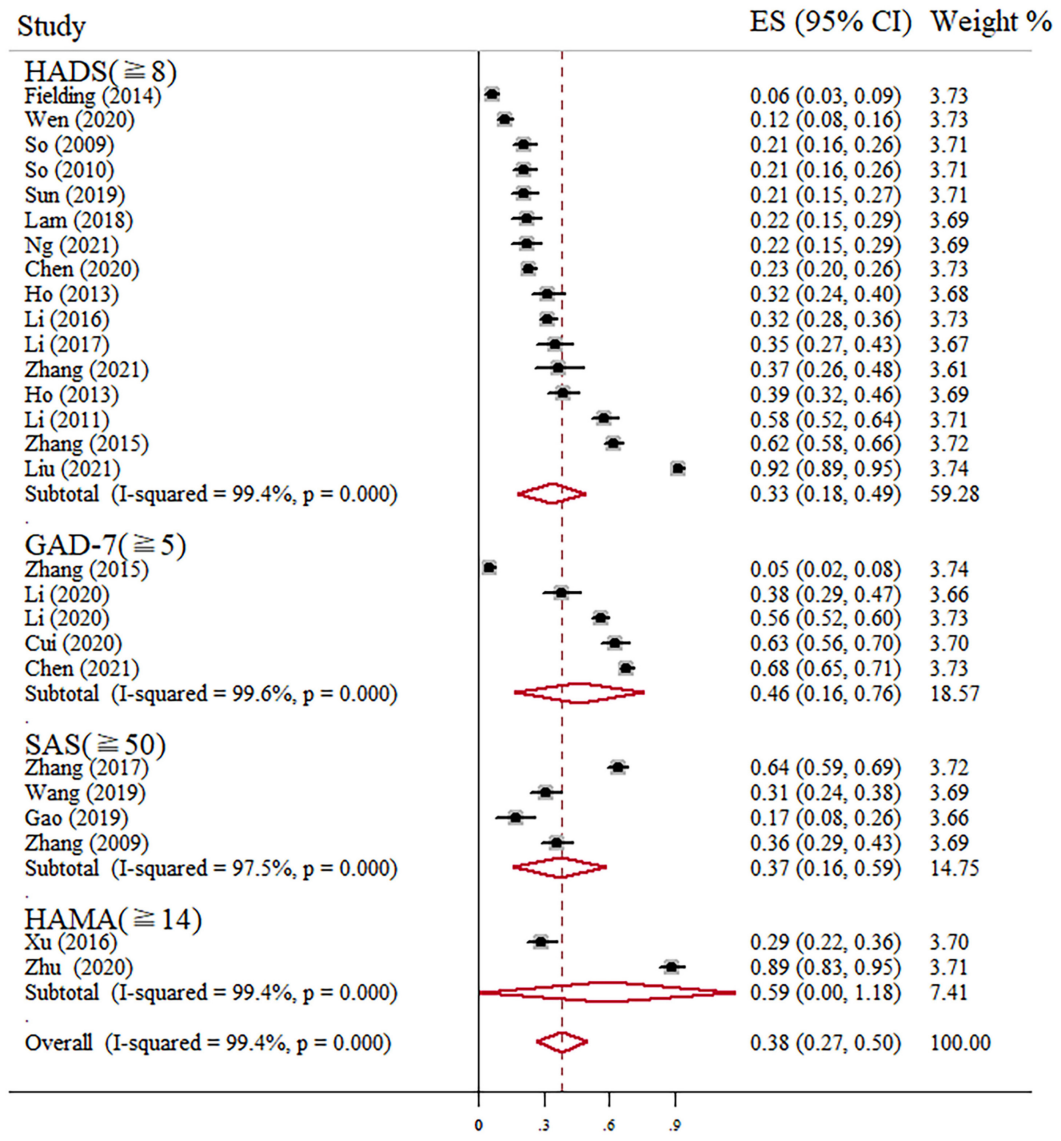
Forest plot of prevalence of anxiety among Chinese BC patients.

**Figure 3:**
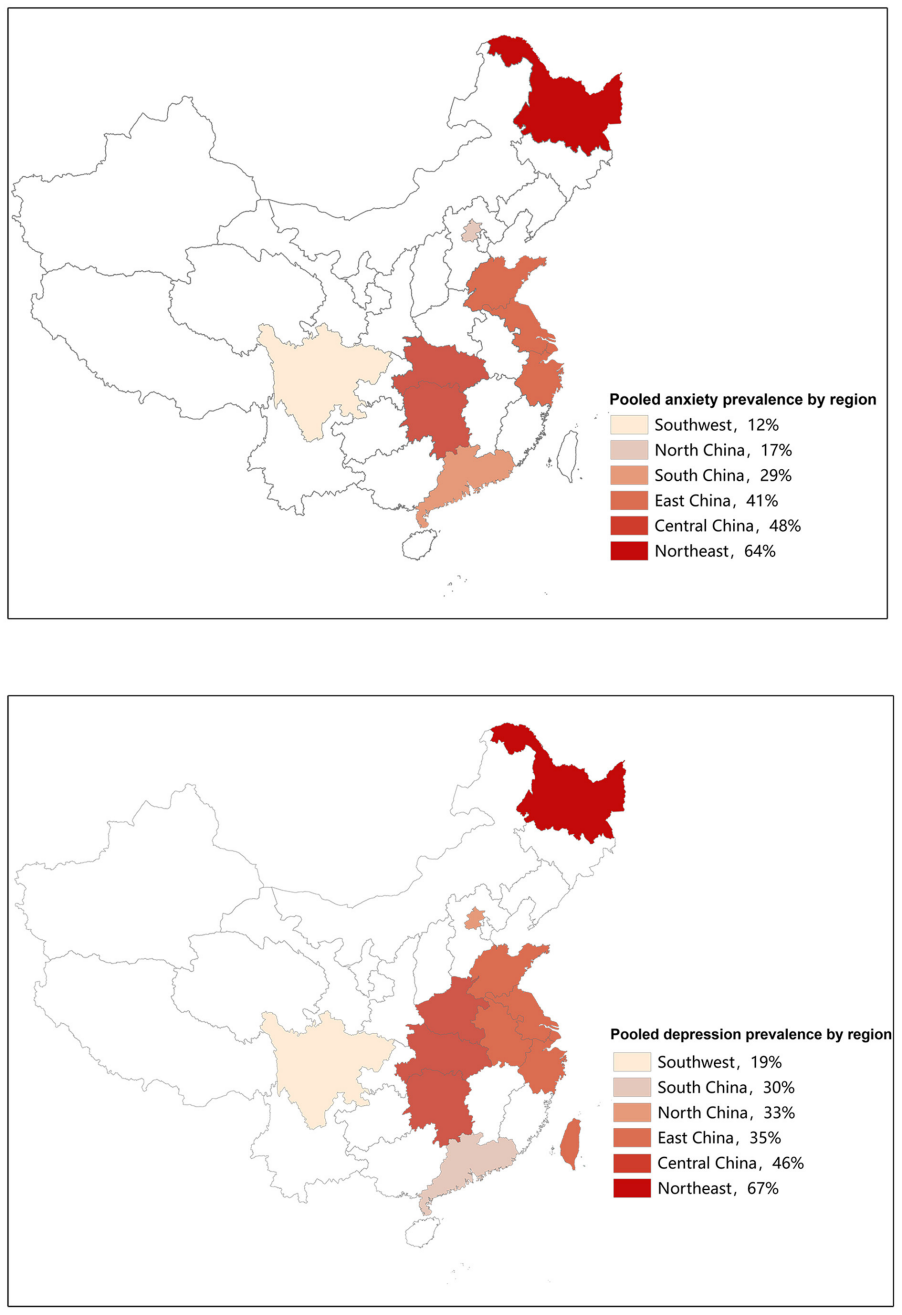
The prevalence of anxiety and depression by regions.

Prevalence of anxiety ranged from 5 to 92% in independent studies (Table 1). We found that the pooled prevalence of anxiety among Chinese BC women was 38% (95% CI, 27–50%, *I*^2^ = 99.4%) with random-effects model. The summary of meta-analyses and heterogeneity assessments were indicated in Table 2. Meta-analyses pooled the prevalence of depression to be 33% (95% CI, 18–49%; *I*^2^ = 99.4%, *p* < 0.001) and 46% (95% CI, 16–76%; *I*^2^ = 99.6%, *p* < 0.001) according to the HADS with thresholds of 8 and the GAD-7 with thresholds of 5, respectively (Figure 2). Subgroup differences were also found in different regions (*p* < 0.001). The estimated prevalence of anxiety symptoms was higher in the northeast region in this study (64%, 95% CI, 34–94%; Figure 3).

**Table 2:** Methods of detecting anxiety and summary of prevalence and heterogeneity findings.

**Tool**	**Definition/cutoff**	**No. of studies**	**No. of participants**	**Prevalence, % (95% CI)**	**Heterogeneity *I*^2^, %**
HADS	≧8	16	4,279	24 (17, 30)	97.0
	≧9	1	128	53 (44, 62)	-
	≧11	6	1,587	25 (4, 46)	99.4
	≧15	2	396	2 (1, 3)	0.0
GAD-7	≧5	5	2,083	38 (16, 61)	99.3
	≧10	5	1,970	12 (9, 16)	79.0
	≧15	3	1,606	6 (2, 9)	86.8
SAS	≧50	4	794	26 (17, 35)	87.9
	≧60	2	566	18 (2, 34)	95.9
	≧70	2	566	7 (4, 10)	46.2
HAMA	≧8	1	315	55 (50, 61)	-
	≧14	2	2,185	59 (1, 118)	99.4
STAI-S	≧48	1	65	14 (6, 22)	-
STAI-T	≧49	1	65	14 (6, 22)	-

HADS, the Hospital Anxiety and Depression; GAD, Generalized Anxiety Disorder Questionnaire; SAS, Self-Rating Anxiety Scale; HAMA, Hamilton Anxiety Scale; STAI-S, State-Trait Anxiety Inventory-State; STAI-T, State-Trait Anxiety Inventory-Trait.

**Figure 4:**
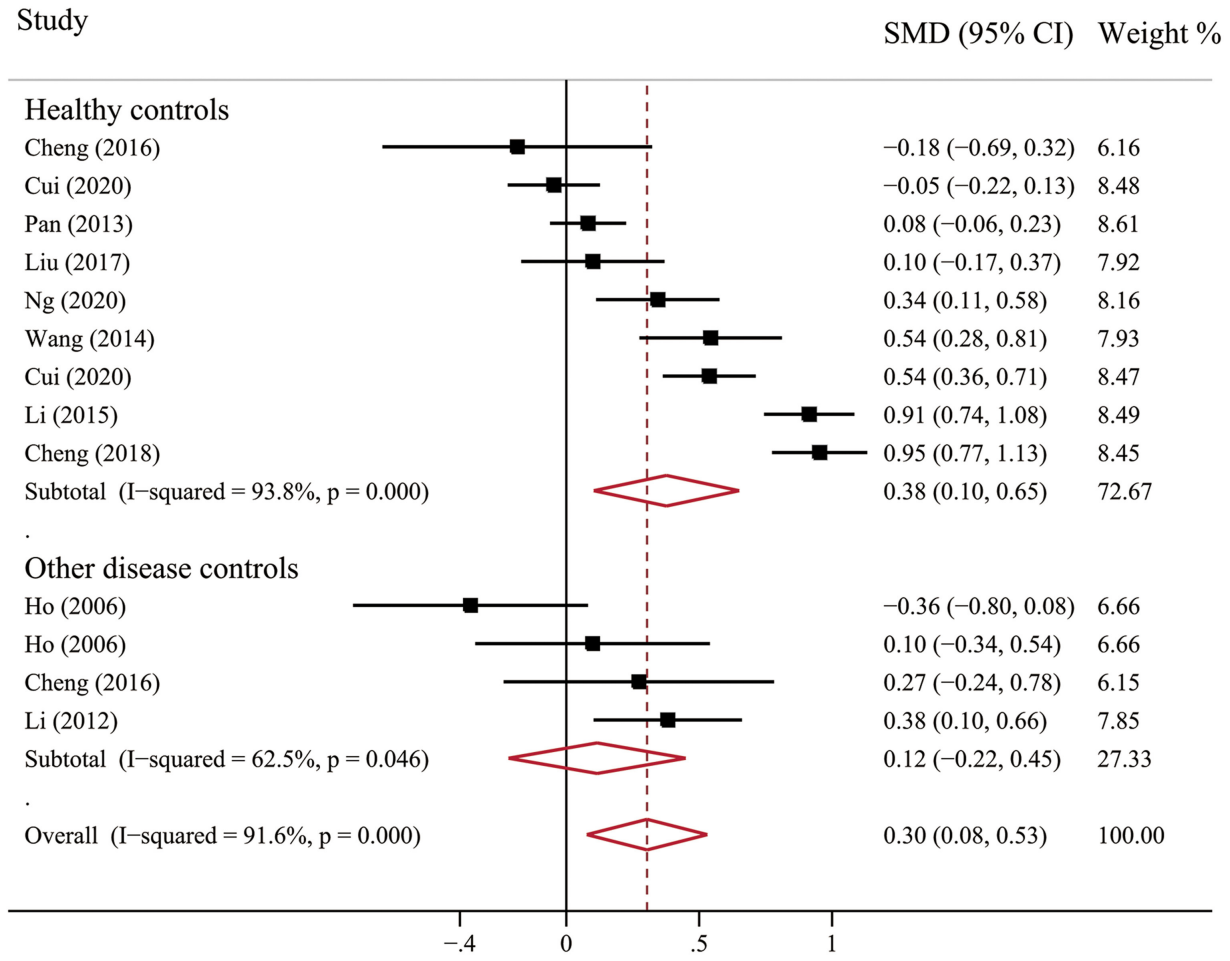
Forest plot of scale scores of anxiety in Chinese BC patients compared with controls groups.

Scale scores of anxiety in Chinese BC patients were compared with control groups in 10 studies: nine with comparison to healthy groups and three with comparison to other diseases (e.g., musculoskeletal condition, colorectal cancer, and nasopharyngeal cancer). Notably, however, studies compared with healthy controls (SMD = 0.38, 95% CI, 0.10–0.65, *I*^2^ = 93.8, *p* < 0.001) showed higher levels of anxiety in BC patients. It's worth emphasizing that there is no significant difference between BC patients and other diseases patients (Figure 4).

### Prevalence and severity of depression among Chinese BC patients

**Figure 5:**
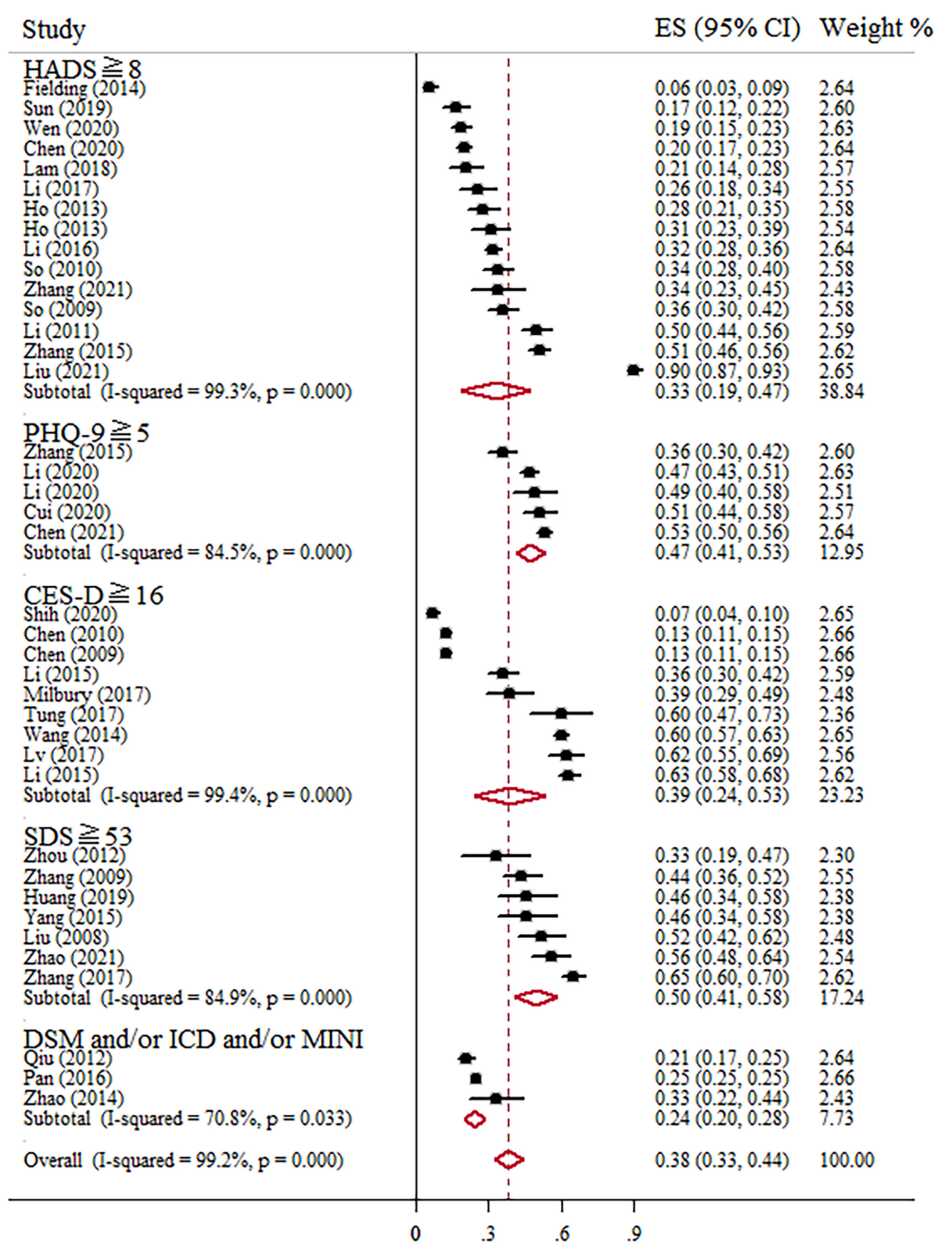
Forest plot of prevalence of depression among Chinese BC patients.

Prevalence of depression ranged between 6 and 90% in independent studies (Table 1). Fifty-eight articles assessed the prevalence of depression, and the pooled prevalence was 38% (95% CI, 33–44%; *I*^2^ = 99.2%, *p* < 0.001) based on the random-effects model. The summary of meta-analyses and heterogeneity assessments were indicated in Table 3. The pooled prevalence of depression based on assessment tools was the lowest in the DSM, ICD or MINI (24%, 95% CI, 20–28%, *I*^2^ = 70.8%, *p* = 0.033) and highest in SDS with cutoff of 53 (50%, 95% CI, 41–58%, *I*^2^ = 84.9%, *p* < 0.001) through sub-group analysis (Figure 5). Subgroup differences were also found in study regions (*p* < 0.001). Consistently, the prevalence estimate of depression was higher as well in the northeast region (including Heilongjiang) in this meta-analysis (67%; 95% CI, 42–91%; Figure 3).

**Table 3:** Methods of detecting depression and summary of prevalence and heterogeneity findings.

**Tool**	**Definition/cutoff**	**No. of studies**	**No. of participants**	**Prevalence, % (95% CI)**	**Heterogeneity *I*^2^, %**
HADS	≧8	15	4,139	24 (18, 30)	95.6
	≧9	1	128	28 (20, 36)	-
	≧11	6	1,587	30 (10, 50)	99.2
	≧15	1	215	3 (1, 5)	-
PHQ-9	≧5	5	2,197	38 (29, 46)	93.7
	≧8	1	114	20 (13, 27)	-
	≧10	3	1,601	16 (11, 20)	77.1
	≧15	4	1,861	8 (5, 10)	71.5
CES-D	≧10	2	2,799	13 (12, 14)	0.0
	≧16	9	5,176	37 (25, 49)	99.2
	≧27	3	1,694	29 (16, 42)	96.6
DSM and/or ICD and/or MINI for anxiety disorder		3	39,952	24 (21, 28)	70.8
SDS	≧50	1	176	34 (27, 41)	-
	≧53	7	994	45 (38, 52)	78.2
	≧60	1	410	26 (22, 30)	-
	≧63	3	554	25 (16, 33)	69.9
	≧73	2	452	5 (3, 7)	0.0
BDI	≧5	1	505	41 (37, 45)	-
	≧12	1	23	48 (28, 68)	-
	≧14	1	205	23 (17, 29)	-
	≧20	1	205	15 (10, 20)	-
HAMD	≧8	1	205	6 (3, 9)	-
	≧17	1	315	47 (42, 53)	-
	>20	1	575	2 (0, 4)	-
PSSG		1	582	27 (23, 31)	-

HADS, the Hospital Anxiety and Depression Scale; PHQ, Patient Health Questionnaire; CES-D, the Center for Epidemiologic Studies Depression Scale; DSM, Diagnostic and Statistical Manual of Mental Disorders; ICD, International Classification of Diseases; MINI, The Mini-International Neuropsychiatric Interview; SDS, Self-Rating Depression Scale; BDI, Beck depression rating scale; HAMD, Hamilton Depression Scale; PSSG, Psychosocial Stress Survey for Groups.

**Figure 6:**
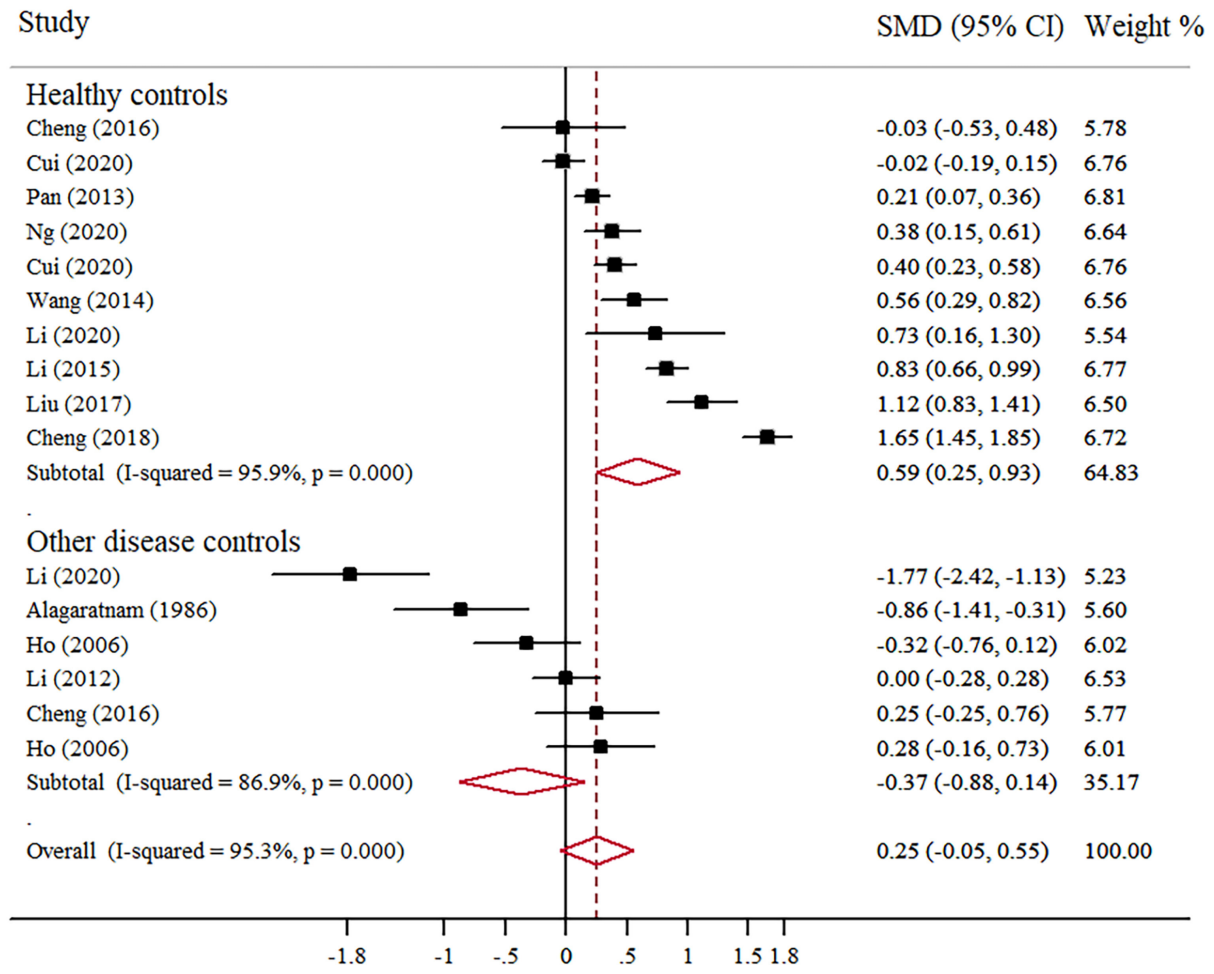
Forest plot of scale scores of depression in Chinese BC patients compared with controls groups.

Mean scores of depression in Chinese BC patients were compared with control groups in 12 studies: 10 with comparison to healthy groups and five with comparison to other diseases (e.g., depressive disorders, musculoskeletal condition, colorectal cancer, nasopharyngeal cancer, and other cancers). According to scale scores, depression in BC patients was higher than that in healthy controls (SMD = 0.59; 95% CI, 0.25–0.93; *I*^2^ = 95.9%, *p* < 0.001), but not significantly different from patients with other diseases (Figure 6).

### Sensitivity analysis and publication bias

Using the sensitivity analysis, no individual study influences the results, for the corresponding pooled ES and SMD were not significantly altered (Supplementary material 4). According to Egger's test, there is no significant publication bias in studies above, including the prevalence and score of anxiety (Supplementary material 5).

## Discussion

The overall prevalence of anxiety and depression was 38% (range: 27–50%) and 38% (range: 33–44%) with a total of 63 studies involving 53,513 Chinese adult BC patients in our meta-analysis, indicating that anxiety and depression also did coexist in Chinese BC cancer patients. This finding is worth noting because anxiety and depression comorbidity tend to have more severe symptoms, higher treatment costs, and worse prognosis than people with a single disorder. What's more, these prevalence estimates were relatively higher than the general population and other cancers in China (29–33). Possibly because BC is considered as a scary disease, with a negative effect on women's self-image and sexual relations, it is reasonable to find a higher prevalence of anxiety/depression in BC patients.

According to the results of a systematic review, the global prevalence of anxiety among BC patients was 41.9% (19). Conversely, another meta-analysis claimed that the prevalence of long-term symptoms of anxiety after BC treatment was 27.2% (34). Such discrepancy could be explained by the differences in the stage of disease and tools used for assessing anxiety. Our study showed that the prevalence of depression among Chinese BC patients is obviously different from the rates that have been reported worldwide (16, 20, 35). The observed differences from each country are likely due to differences in cultural, behavioral, methodological variations, and demographic characteristics, including the economic status of the population, age, social and family support, education, and marital status. Because the development of anxiety and depression could result in worse functional rehabilitation, decreased quality of life, and a higher risk of mortality among BC patients, these findings suggest that immediate attention should be paid to this population. Furthermore, longitudinal studies are needed to understand the trajectory of anxiety and depression among BC patients, from diagnosis to treatment and survivorship. This can help identify critical time points for intervention and inform healthcare providers on how to better support patients throughout their cancer journey.

However, simply reporting the prevalence of anxiety and depression in Chinese BC patients is not enough, the use of a comparable control group is essential to facilitate the reliability and accuracy of the levels of anxiety and depression in BC patients. The level of anxiety (SMD = 0.38, 95% CI, 0.10–0.65) and depression (SMD = 0.59, 95% CI, 0.25–0.93) were significantly higher in patients with BC than in healthy controls, but there are no statistical differences compared with other disease groups. This is the first meta-analysis reporting anxiety and depression in Chinese BC patients compared with those control groups. On the whole, our findings were similar to the results from previous studies conducted in the general Chinese population (36–39). Possible reasons for no difference between BC and other disease patients may be the variations in the composition of disease, including different types of cancer and depressive disorders. Some of them had a higher level of anxiety and depression than BC patients, while some were lower, which caused the mixed results. Another reason is the assessment tools used by individual studies were rather heterogeneous, and the mean scores are quite different. Future studies may consider pooling the prevalence of anxiety and depression with a uniform instrument to compare BC and a single disease.

However, according to the overall forest plots in Figures 2, 5, substantial heterogeneity (*I*^2^ = 99.4%, *p* < 0.001; *I*^2^ = 99.2%, *p* < 0.001) was observed among the studies. Therefore, we performed subgroup analysis and sensitivity analysis to analyze the sources of heterogeneity. Our sensitivity analyses showed that the anxiety and depression prevalence estimates were stable and no study needs to be excluded. Given the effect of regions on the prevalence of anxiety and depression in BC, we performed a stratified meta-analysis based on the regional groups and found that it contributed importantly to the observed heterogeneity. Studies conducted in the northeast region had remarkably higher anxiety and depression prevalence estimates than other districts in China. Cultural traditions and economic status may explain the observed differences. In northeast China, there is the highest incidence and mortality of BC partly due to the low fertility rate and salty eating habits (40–42). In addition, because of the under-developed economic status and insufficient health resource allocation, many patients were already in the middle-advanced stage when definitely diagnosed with BC, which will greatly increase the level of anxiety and depression. Nevertheless, fewer studies conducted in North China and southwest regions were included in this meta-analysis. Therefore, future original studies are needed to investigate the levels of anxiety and depression among BC patients in these regions.

In this meta-analysis, many diagnostic criteria were used for data extraction and synthesis, and the summary prevalence varies by different criteria. The prevalence of anxiety is up to 59% when using HAMA for assessment, while only 33% by HADS. Further, it even doubles for prevalence estimate of depression when using different tools. Although these studies used standardized scales, such as the HADS, GAD-7, or PHQ-9, the best cut-point for screening tools in BC patients have not been determined, and several cut-off scores were used in many studies. Additionally, the prevalence was often overestimated because screening tools prefer to give priority to sensitivity rather than specificity (43). Diagnostic interviews using DSM, MINI, or ICD criteria were considered as gold standard method, while they were often time-consuming and expensive. Thus, in a busy hospital environment, self-report screening tools were more feasible than diagnostic interviews for easier to fill out, cheaper to use. As a result, it indicated that future studies should use the general tools and cut-points especially for BC patients, and try to screen for anxiety and depression in clinical practice if possible.

The current study also has some limitations that need to be pointed out. Firstly, high level of heterogeneity between studies. The varying sample characteristics may contribute to the heterogeneity, such as working status, disease stages, treatment strategies, and evaluate time nodes, which could not be extracted for analysis, leaving substantial heterogeneity between studies largely unexplained by the variables studied. Secondly, publication bias, we searched the literature only in Chinese and English language which limited access to unpublished results. Although Egger's test showed that there was no publication bias, more studies are still needed to verify our results. Thirdly, there are differences in ethnicity and regions. The included studies were conducted in 17 out of the 34 provinces/municipalities/autonomous regions in China, and most studies were conducted in developed urban cities, limiting the generalizability of the findings. Therefore, cross-regional studies and studies focusing on northwest, southwest regions, and rural areas of China are needed to contribute to a more comprehensive understanding of mental health problems among Chinese BC women.

## Conclusion

This meta-analysis revealed the high prevalence and severity of anxiety and depression among BC patients in China, indicating that it has become a major health issue nationally. It is worthwhile to provide resources for reducing social stigma and changing public perceptions of emotional distress. Furthermore, healthcare providers should identify and support BC patients experiencing anxiety and depression, such as routine mental health screening, referral to mental health services, and incorporating psychological interventions as part of the overall treatment plan. The results also showed that the prevalence of anxiety and depression varies in different regions of China, with the highest level in the northeast. Therefore, targeted policies and measures should be distinguished and implemented for different regions.

## Data availability statement

The original contributions presented in the study are included in the article/Supplementary material, further inquiries can be directed to the corresponding authors.

## Author contributions

FT and ZL: conception and design of the research and drafting the manuscript. FT, MX, QZ, and LT: acquisition of data. LT and QZ: data extraction and quality evaluation. FT and MX: statistical analysis. ZL and JF: revision of the manuscript for important intellectual content. All authors have read and approved the final manuscript.
