# Supplementary material for: Prevalence and severity of anxiety and depression in Chinese patients with breast cancer: a systematic review and meta-analysis
Source: Front Psychiatry. 2023 Jun 28;14:1080413. doi: 10.3389/fpsyt.2023.1080413 (PMC10336240; doi:10.3389/fpsyt.2023.1080413)
Supplement: Supplementary file 1 [file Data_Sheet_1.pdf]

Supplementary Information 1

Search strategy of PubMed (The retrieval time: 20210729)

Search	Query	Items found
#1	("anxiety disorder*"[Title/Abstract] OR "Anxiety"[Title/Abstract] OR "Anxiety"[MeSH Terms] OR "anxiety disorder*"[MeSH Terms])	284923
#2	("Depression"[Mesh] OR "Depressive Disorder"[Mesh]) OR (depress*[Title/Abstract]) OR ("depress* disorder*"[Title/Abstract]) OR (depressive symptom*[Title/Abstract])	539610
#3	("dysthymi*"[Title/Abstract] OR "affective symptom*"[Title/Abstract] OR "adjustment disorder*"[Title/Abstract] OR "mood disorder*"[Title/Abstract] OR "affective disorder*"[Title/Abstract] OR "Dysthymic Disorder"[MeSH Terms] OR "Affective Symptoms"[MeSH Terms] OR "Adjustment Disorders"[MeSH Terms] OR "Mood Disorders"[MeSH Terms])	166347
#4	("breast neoplasms"[MeSH Terms] OR ("breast"[All Fields] AND "neoplasms"[All Fields]) OR "breast neoplasms"[All Fields] OR ("breast"[All Fields] AND "cancer"[All Fields]) OR "breast cancer"[All Fields])	402563
#5	（"China"[Affiliation] OR "chinese"[Affiliation] OR "China"[Title/Abstract] OR "chinese"[Title/Abstract] OR "China"[MeSH Terms]）	2132995
#6	#1 OR #2 OR #3	727312
#7	#4 AND #6	6648
#8	#5 AND #7	383

Search strategy of Web of Science (The retrieval time: 20210729)

Search	Query	Items found
#1	(TS=("anxiety disorder*")) OR TS=(Anxiety)	230383
#2	((((TS=(Depression)) OR TS=("depressive Disorder")) OR TS=(depress*)) OR TS=("depress* disorder*")) OR TS=("depressive symptom*")	516398
#3	((((TS=(dysthymi*)) OR TS=("affective symptom*")) OR TS=("adjustment disorder*")) OR TS=("mood disorder*")) OR TS=("affective disorder*")	41893
#4	(TS=("breast neoplasms")) OR TS=(("breast cancer"))	486321
#5	((((TS=(China)) OR TS=(chinese)) OR AD=(China)) OR AD=(chineses)) NOT (SILOID==("PPRN"))	2040635
#6	#1 OR #2 OR #3	628509
#7	#4 AND #6	9015
#8	#5 AND #7	497

Search strategy of Cochrane Library (The retrieval time: 20210729)

Search	Query	Items found
#1	("anxiety disorder*"):ti,ab,kw OR (anxiety):ti,ab,kw	69599
#2	("depression disorder*"):ti,ab,kw OR (depression):ti,ab,kw OR ("depress* disorder*"):ti,ab,kw OR ("depressive symptom*"):ti,ab,kw OR (depress*):ti,ab,kw	107643
#3	(dysthymi*):ti,ab,kw OR ("affective symptom*"):ti,ab,kw OR ("adjustment disorder*"):ti,ab,kw OR ("mood disorder*"):ti,ab,kw OR ("affective disorder*"):ti,ab,kw	8008
#4	("breast neoplasms"):ti,ab,kw OR ("breast cancer"):ti,ab,kw	42607
#5	("China"):ti,ab,kw OR (chinese):ti,ab,kw	48476
#6	#1 OR #2 OR #3	142165
#7	#4 AND #6	2945
#8	#5 AND #7	88

Search strategy of CINAHL (The retrieval time: 20210729)

Search	Query	Items found
#1	(TS=("anxiety disorder*")) OR TS=(Anxiety)	230383
#2	((((TS=(Depression)) OR TS=("depressive Disorder")) OR TS=(depress*)) OR TS=("depress* disorder*")) OR TS=("depressive symptom*")	516398
#3	((((TS=(dysthymi*)) OR TS=("affective symptom*")) OR TS=("adjustment disorder*")) OR TS=("mood disorder*")) OR TS=("affective disorder*")	41893
#4	(TS=("breast neoplasms")) OR TS=(("breast cancer"))	486321
#5	((((TS=(China)) OR TS=(chinese)) OR AD=(China)) OR AD=(chineses)) NOT (SILOID==("PPRN"))	2040635
#6	#1 OR #2 OR #3	628509
#7	#4 AND #6	9015
#8	#5 AND #7	79

Search strategy of Embase (The retrieval time: 20210729)

Search	Query	Items found
#1	("anxiety disorder*" or anxiety).ab.	14547
#2	(Depression or "depressi* Disorder" or depress* or "depressive symptom*").ab.	36477
#3	(dysthymi* or "affective symptom*" or "adjustment disorder*" or "mood disorder*" or "affective disorder*").ab.	2128
#4	("breast neoplasms" or "breast cancer").ab.	34213
#5	(china or chinese).ab. or china.in. or chinese.in.	107881
#6	#1 OR #2 OR #3	43887
#7	#4 AND #6	7893
#8	#5 AND #7	537

Search strategy of Scopus (The retrieval time: 20210729)

Search	Query	Items found
#1	(TITLE-ABS-KEY ("anxiety disorder*") OR TITLE-ABS-KEY (anxiety))	514655
#2	(TITLE-ABS-KEY (depression) OR TITLE-ABS-KEY ("depressive disorder") OR TITLE-ABS-KEY (depress*) OR TITLE-ABS-KEY ("depress* disorder*") OR TITLE-ABS-KEY ("depressive symptom*"))	1058446
#3	(TITLE-ABS-KEY (dysthymi*) OR TITLE-ABS-KEY ("affective symptom*") OR TITLE-ABS-KEY ("adjustment disorder*") OR TITLE-ABS-KEY ("mood disorder*") OR TITLE-ABS-KEY ("affective disorder*"))	110674
#4	(TITLE-ABS-KEY (breast AND neoplasms) OR TITLE-ABS-KEY (breast AND cancer))	666563
#5	(TITLE-ABS-KEY (china) OR AFFILCOUNTRY (china) OR TITLE-ABS-KEY (chinese) OR AFFIL (chinese))	10455976
#6	#1 OR #2 OR #3	1368476
#7	#4 AND #6	9023
#8	#5 AND #7	670

Search strategy of PsycINFO (The retrieval time: 20210729)

Search	Query	Items found
#1	Abstract: "anxiety disorder*" OR MeSH: anxiety OR Abstract: anxiety OR MeSH: "anxiety disorder*"	241767
#2	MeSH: "Depression" OR MeSH: "Depressive Disorder" OR Abstract: Depressi* OR Abstract: "depress* disorder*" OR Abstract: depressive symptom*	354608
#3	Abstract: dysthymi* OR Abstract: "affective symptom*" OR Abstract: "adjustment disorder*" OR Abstract: "mood disorder*" OR Abstract: "affective disorder*" OR MeSH: "Dysthymic Disorder" OR MeSH: "Affective Symptoms" OR MeSH: "Adjustment Disorders" OR MeSH: "Mood Disorders"	53059
#4	MeSH: "breast neoplasms" OR Abstract: breast neoplasms OR Abstract: breast cancer	17259
#5	Abstract: China OR Affiliation: China OR Affiliation: chinese OR Abstract: chinese	167438
#6	#1 OR #2 OR #3	511320
#7	#4 AND #6	2920
#8	#5 AND #7	88

Search strategy of CNKI/ (The retrieval time: 20210729)

Search Query	Items found
(主题=焦虑 + 抑郁 + 情感障碍 + 情绪障碍) AND (主题=乳腺癌 + 乳房癌 + 乳癌 + 乳腺肿瘤) 来源类别：北大核心,CSSCI,CSCD	317

Search strategy of Wanfang (The retrieval time: 20210729)

Search Query	Items found
主题:(乳腺癌 OR 乳房癌 OR 乳癌 OR 乳腺肿瘤) and 主题:(焦虑 + 抑郁 + 情感障碍 + 情绪障碍)	1655

Search strategy of SinoMed (The retrieval time: 20210729)

Search	Query	Items found
#1	"焦虑"[加权:扩展] OR "焦虑"[常用字段:智能]	228616
#2	"抑郁"[常用字段:智能] OR "抑郁"[加权:扩展]	175563
#3	"情感障碍"[加权:扩展] OR "情绪障碍"[加权:扩展] OR "情感障碍"[常用字段:智能] OR "情绪障碍"[常用字段:智能]	64654
#4	"乳腺癌"[常用字段:智能] OR "乳腺肿瘤"[常用字段] OR "乳腺肿瘤"[加权:扩展] OR "乳癌"[常用字段:智能] OR "乳房癌"[常用字段:智能]	132770
#5	#1 OR #2 OR #3	314810
#6	#4 AND #5	1042
#7	(#6) AND ("2"[期刊类型])	200
