# Supplementary material for: Prevalence and severity of anxiety and depression in Chinese patients with breast cancer: a systematic review and meta-analysis
Source: Front Psychiatry. 2023 Jun 28;14:1080413. doi: 10.3389/fpsyt.2023.1080413 (PMC10336240; doi:10.3389/fpsyt.2023.1080413)
Supplement: Supplementary file 2 [file Data_Sheet_2.pdf]

Supplementary Information 2

The included studies in the present systematic review and meta-analysis

1. Zhao TT, Ming Y, Zhang XJ. A longitudinal study on depression in young and middle-aged patients and on their spouses' psychological response and intimate relationship. *Journal of Nursing Science*. (2021) 36(23):75-78. DOI:10.3870/j. issn.1001-4152. 2021.23.075
2. Zhu LJ, Cheng YH, Li XN, Jiang XY, Yu QQ. Psychological analysis of breast cancer patients with coronaavirus disease 2019. *Chinese Journal of Nursing*. (2020) 55(S1):377-379.
3. Zhou YH, Pang Y, Han XK, He SZ, Li JJ, Tang LL. Death anxiety and related factors in young women with advanced breast cancer. *Chinese Mental Health Journal*. (2020) 34(11):891-897. doi: 10.3969/j. issn.1000-6729. 2020.11.002
4. Wen M, Xu HP, Zhang XL, Wang Hao, Yang J, Li Y, et al. Investigation of the delay of medical treatment and anxiety and depression of breast cancer patients during the pandemic of COVID-19. *Modern Preventive Medicine*. (2020) 47(22):4093-4097.
5. Chen DM, Zhang Y, Lv LY, Zhang J, Zhang L, Wan DZ. A survey of treatment and psychological status of breast cancer patients during the pandemic of COVID-19. *MODERN ONCOLOGY.* (2020) 28(12):2179-2182. DOI: 10.3969/j.issn.1672-4992.2020.12.041
6. Gao C, Chen JY, He Y, Pang Y, Tang LL. Relationship between anxiety symptom and radiotherapy set-up errors in patients with early breast cancer. *Chinese Mental Health Journal.* (2019) 33(11):812-815. doi: 10.3969/j. issn.1000-6729. 2019.11.003
7. Lv LM, Zhang XH, Wang XX, Zhang JL. A study on subthreshold depression and social participation among young and middle-aged patients after breast cancer surgery. *Journal of Nursing Science.* (2017) 32(6):84-87. DOI: 10.3870 /j.issn.1001-4152.2017.06.084
8. Liu SX, Yang Q, Feng DL, Wang Q, Chen Y, Zheng SJ, et al. Study on the status quo of working status and quality of life of female breast cancer patients after discharge. *J Sichuan Univ (Med Sci Edi)*. (2017) 48(4):614-617. DOI：10.13464/j.scuxbyxb.2017.04.022
9. Xu HP, Chu JH, Q C, Han W, Yang XW, Tang HH, et al. Analysis of relation between anxiety and understanding social support and living standard during endocrine therapy for breast cancer patients. *CHIN J CANCER PREV TREAT.* (2016) 23(14):909-912. DOI: 10.16073/j.cnki.cjcpt.2016.14.002
10. Yang MH, Cheng HD, Chen ZD, Huang ZL, Wang XL. The impact of depression on chemotherapy induced cognitive impairment in breast cancer patients. *Acta Universitatis Medicinalis Anhui.* (2015) 50(5):645-647. DOI:10.19405/j.cnki.issn1000-1492.2015.05.020
11. Zhang LJ, Xin MZ, Yan J, Wu Y, Sun KL, Liao WMF, et al. The correlations between social supports and psychological pressure, anxiety and depression among patients preliminarily diagnosed with breast cancer. *The Journal of Practical Medicine.* (2015) 31(21):3601-3605. Doi:10.3969/j.issn.1006-5725.2015.21.045
12. Xu N, Zhang PY, Zhou DX, Liu CH, Zhang YY, Wang YY. Investigation of depression status in postoperation patients with breast cancer and analysis of its relationship with risk factors. *China Journal of Modern Medicine.* (2015) 25(34):68-72.
13. Li LY, Zhu XZ, Wang YP, Yang YL, Zhang JQ. A Prospective Study of Emotional Suppression and Depressive Symptoms in Women with Breast Cancer. *Chinese Journal of Clinical Psychology.* (2015) 23(5):915-918. DOI: 10.16128/j.cnki.1005-3611.2015.05.036

14. Xu LX, Shen SS, He JJ, Fu Y, Xue XD, Liang Y, et al. Relationship among anxiety，depression，well-being index and social support in breast cancer patients. *Chinese Mental Health Journal.* (2013) 27(6): 473-478. doi:10.3969 /j.issn.1000-6729. 2013. 06. 014
15. Pang Y, FISCHER I, KOCH M, FRITZSCHE K, TANG LL. Psychosomatic symptoms and quality of life in breast cancer patients. *Chinese Mental Health Journal.* (2013) 27(4):257-261. doi:10.3969 /j.issn.1000-6729. 2013. 04. 005
16. Zhou HG, Liu SZ, Sun HW. The Relationship among Depression , Locus of Control and Cognitive Attitude of Patients with Breast Cancer. *China Journal of Health Psychology.* (2012) 20(8):1149-1151. DOI:10.13342/j.cnki.cjhp.2012.08.046
17. Zhang J, Chen L, Wang P, Wu ZH, Liu XR, Li HL, et al. Study on the correlation between hope level and anxiety and depression in breast cancer patients. *Chinese Journal of Practical Surgery.* (2009) 30(5):394.
18. Liu XF, Ou HX, Li P. The Relationship Between Depression and Coping Modes in Patient with Breast Cancer. *ACTA UNIVERSITATIS MEDICINALIS NANJING(Natural Science).* (2008) 28(8):1058-1060.
19. Cheng ASK, Lau LOC, Ma YNH, Ngai RH, Fong SSL. Impact of Cognitive and Psychological Symptoms on Work Productivity and Quality of Life among Breast Cancer Survivors in Hong Kong. *Hong Kong J Occup Ther*. (2016) 28(1):15-23. doi: 10.1016/j.hkjot.2016.11.002
20. Ho RT, Fong TC, Chan CK, Chan CL. The associations between diurnal cortisol patterns, self-perceived social support, and sleep behavior in Chinese breast cancer patients. *Psychoneuroendocrinology.* (2013), 38(10), 2337–2342. doi: 10.1016/j.psyneuen.2013.05.004. Epub 2013 Jun 3.
21. Ho RT, Kwan TT, Cheung IK, Chan CK, Lo PH, Yip PS, et al. Association of Fatigue with Perceived Stress in Chinese Women with Early Stage Breast Cancer Awaiting Adjuvant Radiotherapy. *Stress Health.* (2015) 31(3):214-221. doi:10.1002/smi.2548
22. Lam WWT, Ng D, Wong S, Lee TMC, Kwong A, Fielding R. The role of cognitive bias in relation to persistent distress among women diagnosed with breast cancer. *Psychooncology*. (2018) 27(3):983-989. doi:10.1002/pon.4620
23. Li JJ, Cesar AS, Feng HF, Wang LC, Zhang PC, Xu YB, et al. Patient-reported Outcomes of Patients With Breast Cancer During the COVID-19 Outbreak in the Epicenter of China: A Cross-sectional Survey Study. *Clin Breast Cancer*. (2020) 20(5): e651-e662. doi:10.1016/j.clbc.2020.06.003
24. Li J, Zhang R, Li S, Gao W, Zhang H, Bai H, et al. Association of Depressive Symptoms with Perceived Stress and Proinflammatory Cytokines in Treatment-Naïve Patients with Breast Cancer. *Biol Res Nurs.* (2021) 23(2):180-187. doi:10.1177/1099800420941094
25. Li L, Li S, Wang Y, Yi J, Yang Y, He J, et al. Coping Profiles Differentiate Psychological Adjustment in Chinese Women Newly Diagnosed With Breast Cancer. *Integr Cancer Ther.* (2017) 16(2):196-204. doi: 10.1177/1534735416646854. Epub 2016 May 6.
26. Li WW, Lam WW, Au AH, Ye M, Law WL, Poon J, et al. Interpreting differences in patterns of supportive care needs between patients with breast cancer and patients with colorectal cancer. *Psychooncology.* (2013) 22(4):792-798. doi:10.1002/pon.3068
27. Pan XF, Fei MD, Zhang KY, Fan ZL, Fu FH, Fan JH. Psychopathological profile of women with breast cancer based on the symptom checklist-90-R. *Asian Pac J Cancer Prev*. (2014) 14(11):6579-6584. doi:10.7314/apjcp.2013.14.11.6579

28. Shih IH, Lin CY, Fang SY. Prioritizing care for women with breast cancer based on survival stage: A study examining the association between physical symptoms, psychological distress and unmet needs. *Eur J Oncol Nurs*. (2020) 48:101816. doi:10.1016/j.ejon.2020.101816
29. So WK, Marsh G, Ling WM, Leung FY, Lo JC, Yeung M, et al. The symptom cluster of fatigue, pain, anxiety, and depression and the effect on the quality of life of women receiving treatment for breast cancer: a multicenter study. *Oncol Nurs Forum*. (2009) 36(4):E205-E214. doi:10.1188/09.ONF.E205-E214
30. So WK, Marsh G, Ling WM, Leung FY, Lo JC, Yeung M, et al. Anxiety, depression and quality of life among Chinese breast cancer patients during adjuvant therapy. *Eur J Oncol Nurs*. (2010) 14(1):17-22. doi:10.1016/j.ejon.2009.07.005
31. Sun XF, Xia F. Long-term fatigue state in postoperative patients with breast cancer. *Chin J Cancer Res*. (2014) 26(1):12-16. doi:10.3978/j.issn.1000-9604.2014.01.12
32. Tong HY, Lin YH, Kao CC, Wu SF. The Relationships Among Symptom Distress, Posttraumatic Stress Symptoms, and Depression in Patients With Female-specific Cancers. *Cancer Nurs*. (2018) 41(3):181-188. doi:10.1097/NCC.0000000000000479
33. Zhao L, Li X, Zhang Z, Song C, Guo C, Zhang Y, et al. Prevalence, correlates and recognition of depression in Chinese inpatients with cancer. *Gen Hosp Psychiatry*. (2014) 36(5):477-482. doi:10.1016/j.genhosppsych.2014.05.005
34. Chen X, Lu W, Zheng Y, Gu K, Chen Z, Zheng W, et al. Exercise, tea consumption, and depression among breast cancer survivors. *J Clin Oncol*. (2010) 28(6):991-998. doi:10.1200/JCO.2009.23.0565
35. Pan HH, Chu CH, Wu LF, Hsieh PC, Chang KC, Li CY. Predictors for Reconstruction and Mood Disorder Associated With Reconstruction in Patients With Breast Cancer and Mastectomy: A Retrospective Cohort Study. *Medicine (Baltimore)*. (2016) 95(3):e2510. doi:10.1097/MD.0000000000002510
36. Zhang J, Zhou Y, Feng Z, Xu Y, Zeng G. Longitudinal Trends in Anxiety, Depression, and Quality of Life During Different Intermittent Periods of Adjuvant Breast Cancer Chemotherapy. *Cancer Nurs*. (2018) 41(1):62-68. doi:10.1097/NCC.0000000000000451
37. Li L, Yang Y, He J, Yi J, Wang Y, Zhang J, et al. Emotional suppression and depressive symptoms in women newly diagnosed with early breast cancer. *BMC Womens Health*. (2015) 15:91. doi:10.1186/s12905-015-0254-6
38. Milbury K, Kavanagh A, Meng Z, Chen Z, Chandwani KD, Garcia K, et al. Depressive symptoms and positive affect in Chinese and United States breast cancer survivors: a cross-cultural comparison. *Support Care Cancer*. (2017) 25(7):2103-2109. doi:10.1007/s00520-017-3612-0
39. Li Y, Yuan C. Levels of fatigue in Chinese women with breast cancer and its correlates: a cross-sectional questionnaire survey. *J Am Acad Nurse Pract*. (2011) 23(3):153-160. doi:10.1111/j.1745-7599.2010.00591.x
40. Li J, Gao W, Yang Q, Cao F. Perceived stress, anxiety, and depression in treatment-naïve women with breast cancer: a case-control study. *Psychooncology*. (2021) 30(2):231-239. doi:10.1002/pon.5555
41. Li J, Gao W, Yu LX, Zhu SY, Cao FL. Breast-related stereotype threat contributes to a symptom cluster in women with breast cancer. *J Clin Nurs*. (2017) 26(9-10):1395-1404. doi:10.1111/jocn.13698

42. Ho, Fung Ling. The effect of emotional support, negative interpersonal interaction, emotional control, and pain upon depression and anxiety among Chinese cancer patients. *Dissertation Abstracts International.* (2006) 66: 0419-4217.
43. Alagaratnam TT, Kung NY. Psychosocial effects of mastectomy: is it due to mastectomy or to the diagnosis of malignancy?. *Br J Psychiatry.* (1986) 149:296-299. doi:10.1192/bjp.149.3.296
44. Cui Q, Cai Z, Li J, Liu Z, Sun S, Chen C, et al. The Psychological Pressures of Breast Cancer Patients During the COVID-19 Outbreak in China-A Comparison With Frontline Female Nurses. *Front Psychiatry.* (2020) 11:559701. doi:10.3389/fpsyt.2020.559701
45. Guo X, Xu J, Ying E, Yu Z, Sun T. Correlation between hormone receptor status and depressive symptoms in patients with metastatic breast cancer. *Oncotarget*. (2017) 8(31):50774-50781. doi:10.18632/oncotarget.15037
46. Chen X, Wang L, Liu L, Jiang M, Wang W, Zhou X, et al. Factors associated with psychological distress among patients with breast cancer during the COVID-19 pandemic: a cross-sectional study in Wuhan, China. *Support Care Cancer*. (2021) 29(8):4773-4782. doi:10.1007/s00520-021-05994-4
47. Chen X, Zheng Y, Zheng W, Gu K, Chen Z, Lu W, et al. Prevalence of depression and its related factors among Chinese women with breast cancer. *Acta Oncol.* (2009) 48(8):1128-1136. doi:10.3109/02841860903188650
48. Cheng ASK, Zeng Y, Liu X, Liu S, Cheng SWC, Kwok CTT, et al. Cognitive challenges while at work and work output in breast cancer survivors employed in a rapidly evolving economy. *J Cancer Surviv.* (2018) 12(6):753-761. doi:10.1007/s11764-018-0712-x
49. Hong JS, Tian J. Prevalence of anxiety and depression and their risk factors in Chinese cancer patients. *Support Care Cancer.* (2014) 22(2):453-459. doi:10.1007/s00520-013-1997-y
50. Lan B, Jiang S, Li T, Sun X, Ma F. Depression, anxiety, and their associated factors among Chinese early breast cancer in women under 35 years of age: A cross sectional study. *Curr Probl Cancer.* (2020) 44(5):100558. doi:10.1016/j.currproblcancer.2020.100558
51. Liu B, Wu X, Shi L, Li H, Wu D, Lai X, et al. Correlations of social isolation and anxiety and depression symptoms among patients with breast cancer of Heilongjiang province in China: The mediating role of social support. *Nurs Open.* (2021) 8(4):1981-1989. doi:10.1002/nop2.876
52. Ng DWL, Fielding R, Lam WWT. The Generalization of Conscious Attentional Avoidance in Response to Threat Among Breast Cancer Women With Persistent Distress. *Front Psychol.* (2020) 11:589088. doi:10.3389/fpsyg.2020.589088
53. Qiu J, Yang M, Chen W, Gao X, Liu S, Shi S, et al. Prevalence and correlates of major depressive disorder in breast cancer survivors in Shanghai, China. *Psychooncology.* (2012) 21(12):1331-1337. doi:10.1002/pon.2075
54. Wang F, Liu J, Liu L, Wang F, Ma Z, Gao D, et al. The status and correlates of depression and anxiety among breast-cancer survivors in Eastern China: a population-based, cross-sectional case-control study. *BMC Public Health.* (2014) 14:326. Published 2014 Apr 8. doi:10.1186/1471-2458-14-326
55. Wang Y, Yi J, He J, Chen G, Li L, Yang Y, et al. Cognitive emotion regulation strategies as predictors of depressive symptoms in women newly diagnosed with breast cancer. *Psychooncology*. (2014) 23(1):93-99. doi:10.1002/pon.3376
56. Wei M, Guo L, Zhu Y, Guo Y, Yv S, Namassevayam G, et al. Type C Personality and

Depression Among Newly Diagnosed Breast Cancer Patients: The Mediating Role of Sense of Coherence. *Neuropsychiatr Dis Treat.* (2019) 15:3519-3529. Published 2019 Dec 24. doi:10.2147/NDT.S230278

57. Wu F, Howell D, Fang Q, Chen J, Yuan C. Trajectory Patterns and Factors Influencing Self-management Behaviors in Chinese Patients With Breast Cancer. *Cancer Nurs*. (2020) 43(2):E105-E112. doi:10.1097/NCC.0000000000000681
58. Zhang L, Zhang H, Zhong Q, Luo Q, Gong N, Zhang Y, et al. Predictors of Quality of Life in Patients with Breast Cancer-Related Lymphedema: Effect of Age, Lymphedema Severity, and Anxiety. *Lymphat Res Biol.* (2021) 19(6):573-579. doi:10.1089/lrb.2020.0073
59. Zhang Y, Xu H, Wang T, He J, Qiao Y, Wei J, et al. Psychosocial Predictors and Outcomes of Delayed Breast Reconstruction in Mastectomized Women in Mainland China: An Observational Study. *PLoS One.* (2015) 10(12):e0144410. Published 2015 Dec 7. doi:10.1371/journal.pone.0144410
60. Fielding R, Lam WW. Psychosocial and physical outcomes after surgery for breast cancer: a 5-to-6-year follow-up. *Hong Kong Med J.* (2014) 20 Suppl 7:9-12.
61. Huang Z, Zhao J, Ding K, Lv Y, Zhang C, Chao HH, et al. Depression involved in self-reported prospective memory problems in survivors of breast cancer who have received chemotherapy. *Medicine (Baltimore).* (2019) 98(16):e15301. doi:10.1097/MD.0000000000015301
62. Li P, Huang J, Wu H, Fu C, Li Y, Qiu J. Impact of lifestyle and psychological stress on the development of early onset breast cancer. *Medicine (Baltimore).* (2016) 95(50):e5529. doi:10.1097/MD.0000000000005529
63. Wang Z, Han X. Clinical significance of breast-conserving surgery for early breast cancer and its impact on patient life quality of life. *J BUON.* (2019) 24(5):1898-1904.
