# Supplementary material for: Prevalence and severity of anxiety and depression in Chinese patients with breast cancer: a systematic review and meta-analysis
Source: Front Psychiatry. 2023 Jun 28;14:1080413. doi: 10.3389/fpsyt.2023.1080413 (PMC10336240; doi:10.3389/fpsyt.2023.1080413)
Supplement: Supplementary file 3 [file Data_Sheet_3.pdf]

Supplementary Information 3

Risk-of-bias assessment of included studies

Year	Author	Item1	Item2	Item3	Item4	Item5	Item6	Item7	Item8	Item9	Item10	overall risk of study bias
2021	Zhao	No	Yes	No	Yes	Yes	Yes	Yes	Yes	Yes	Yes	low
2020	Zhu	No	Yes	No	Yes	Yes	No	No	Yes	Yes	Yes	moderate
2020	Zhou	No	Yes	No	No	Yes	Yes	Yes	Yes	Yes	Yes	low
2020	Wen	No	Yes	No	Yes	Yes	No	No	Yes	Yes	Yes	moderate
2020	Chen	Yes	Yes	No	Yes	Yes	No	No	Yes	Yes	Yes	low
2019	Gao	No	Yes	No	Yes	Yes	No	Yes	Yes	Yes	Yes	moderate
2017	Lv	No	Yes	No	Yes	Yes	Yes	No	Yes	Yes	Yes	low
2017	Liu	No	Yes	No	Yes	Yes	Yes	No	Yes	Yes	Yes	low
2016	Xu	No	Yes	No	Yes	Yes	No	Yes	Yes	Yes	Yes	low
2015	Yang	No	Yes	No	Yes	Yes	Yes	Yes	Yes	Yes	Yes	low
2015	Zhang	No	Yes	No	Yes	Yes	Yes	No	Yes	Yes	Yes	low
2015	Xu	No	Yes	No	Yes	Yes	No	Yes	Yes	Yes	Yes	low
2015	Li	No	Yes	No	Yes	Yes	No	No	Yes	Yes	Yes	moderate
2013	Xu	No	Yes	No	Yes	Yes	No	Yes	Yes	Yes	Yes	low
2013	Pang	No	Yes	No	Yes	Yes	No	No	Yes	Yes	Yes	Moderate
2012	Zhou	No	Yes	No	Yes	Yes	No	Yes	Yes	Yes	Yes	low
2009	Zhang	No	Yes	Yes	Yes	Yes	No	Yes	Yes	Yes	Yes	low
2008	Liu	No	Yes	No	Yes	Yes	No	No	Yes	Yes	Yes	moderate

2016	Cheng	No	Yes	No	No	Yes	Yes	Yes	Yes	No	Yes	moderate
2013	Ho	No	Yes	Yes	Yes	Yes	Yes	Yes	Yes	No	No	low
2013	Ho	No	Yes	Yes	No	Yes	Yes	Yes	Yes	No	Yes	low
2018	Lam	No	No	Yes	Yes	Yes	Yes	Yes	Yes	No	Yes	low
2020	Li	Yes	Yes	No	Yes	Yes	Yes	Yes	Yes	No	Yes	low
2020	Li	No	Yes	Yes	No	Yes	Yes	Yes	Yes	No	Yes	low
2016	Li	No	Yes	Yes	Yes	Yes	Yes	Yes	Yes	No	Yes	low
2012	Li	No	No	No	Yes	Yes	Yes	Yes	Yes	Yes	Yes	low
2013	Pan	No	No	No	No	Yes	Yes	Yes	Yes	Yes	Yes	moderate
2020	Shih	No	No	No	No	Yes	Yes	Yes	Yes	Yes	Yes	moderate
2009	So	No	Yes	No	Yes	Yes	Yes	Yes	Yes	No	Yes	low
2010	So	No	Yes	No	Yes	Yes	Yes	Yes	Yes	No	Yes	low
2019	Sun	No	Yes	No	Yes	Yes	Yes	Yes	Yes	Yes	Yes	low
2017	Tong	No	No	No	Yes	Yes	Yes	Yes	Yes	Yes	Yes	low
2014	Zhao	No	No	No	Yes	Yes	Yes	Yes	Yes	Yes	Yes	low
2010	Chen	No	Yes	Yes	Yes	Yes	Yes	Yes	Yes	Yes	Yes	low
2016	Pan	Yes	Yes	Yes	Yes	Yes	Yes	Yes	Yes	No	Yes	low
2017	Zhang	No	No	No	Yes	Yes	Yes	Yes	Yes	Yes	Yes	low
2015	Li	No	No	No	Yes	Yes	Yes	Yes	Yes	Yes	Yes	low
2017	Milbury	No	Yes	Yes	Yes	Yes	Yes	Yes	Yes	Yes	Yes	low
2011	Li	No	Yes	No	Yes	Yes	Yes	Yes	Yes	No	Yes	low
2020	Li	No	No	Yes	No	Yes	Yes	Yes	Yes	Yes	Yes	low
2017	Li	No	Yes	Yes	No	Yes	Yes	Yes	Yes	No	Yes	low

2006	Ho	No	No	Yes	No	Yes	Yes	Yes	Yes	Yes	Yes	low
1986	Alagaratnam	No	Yes	Yes	No	Yes	Yes	No	Yes	No	Yes	moderate
2020	Cui	Yes	Yes	Yes	Yes	Yes	Yes	Yes	Yes	Yes	Yes	low
2017	Guo	No	Yes	No	Yes	Yes	Yes	No	Yes	Yes	Yes	low
2021	Chen	Yes	Yes	Yes	No	Yes	Yes	Yes	Yes	Yes	Yes	low
2009	Chen	No	Yes	Yes	Yes	Yes	Yes	Yes	Yes	Yes	Yes	low
2018	Cheng	No	Yes	Yes	No	Yes	Yes	Yes	Yes	No	Yes	low
2014	Hong	No	No	Yes	Yes	Yes	Yes	Yes	Yes	No	Yes	low
2020	Lan	No	No	Yes	Yes	Yes	Yes	Yes	Yes	Yes	Yes	low
2021	Liu	No	No	Yes	Yes	Yes	Yes	Yes	Yes	Yes	Yes	low
2020	Ng	No	No	Yes	No	Yes	Yes	Yes	Yes	No	Yes	moderate
2012	Qiu	No	No	Yes	Yes	Yes	Yes	Yes	Yes	Yes	Yes	low
2014	Wang	No	Yes	Yes	Yes	Yes	Yes	Yes	Yes	Yes	Yes	low
2014	Wang	No	Yes	Yes	Yes	Yes	Yes	Yes	Yes	Yes	Yes	low
2019	Wei	No	No	No	Yes	Yes	Yes	Yes	Yes	Yes	Yes	low
2020	Wu	No	Yes	No	No	Yes	Yes	Yes	Yes	No	Yes	moderate
2021	Zhang	No	No	No	Yes	Yes	Yes	Yes	Yes	No	Yes	moderate
2015	Zhang	No	No	Yes	No	Yes	Yes	Yes	Yes	Yes	Yes	low
2014	Fielding	No	Yes	Yes	Yes	Yes	Yes	No	Yes	No	Yes	low
2019	Huang	No	No	Yes	No	Yes	Yes	No	Yes	Yes	Yes	moderate
2016	Liu	No	No	No	Yes	Yes	Yes	Yes	Yes	No	Yes	moderate
2019	Wang	No	No	Yes	No	Yes	Yes	No	Yes	Yes	Yes	moderate
