# Supplementary material for: Prevalence and severity of anxiety and depression in Chinese patients with breast cancer: a systematic review and meta-analysis
Source: Front Psychiatry. 2023 Jun 28;14:1080413. doi: 10.3389/fpsyt.2023.1080413 (PMC10336240; doi:10.3389/fpsyt.2023.1080413)
Supplement: Supplementary file 4 [file Data_Sheet_4.pdf]

Supplementary Information 4

Results of sensitivity analysis

(a) the prevalence of anxiety; (b)mean score of anxiety; (c)the prevalence of depression; (d)mean score of depression

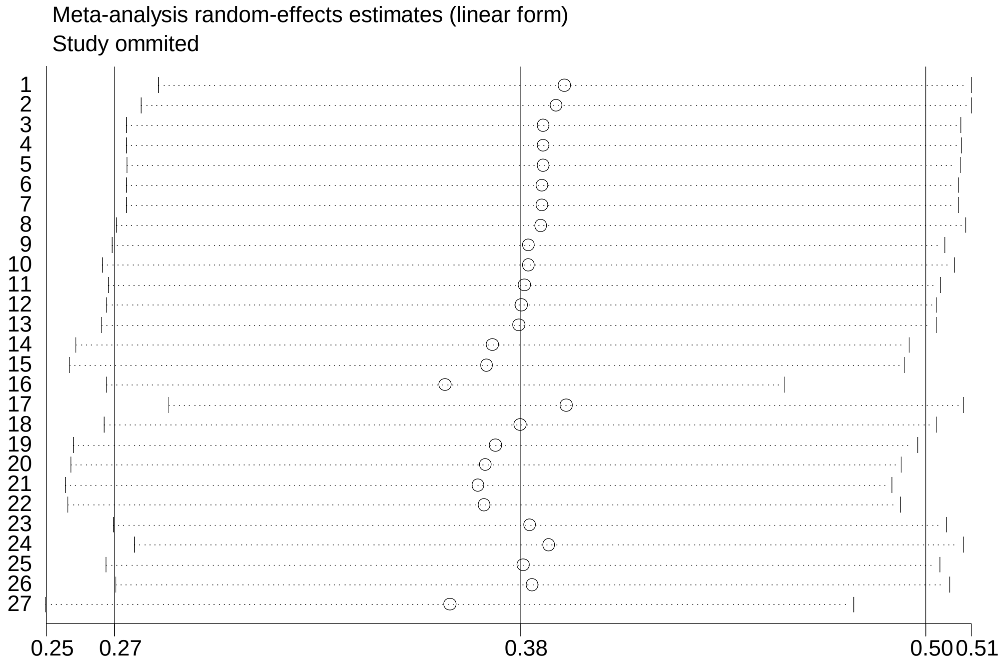

(a)

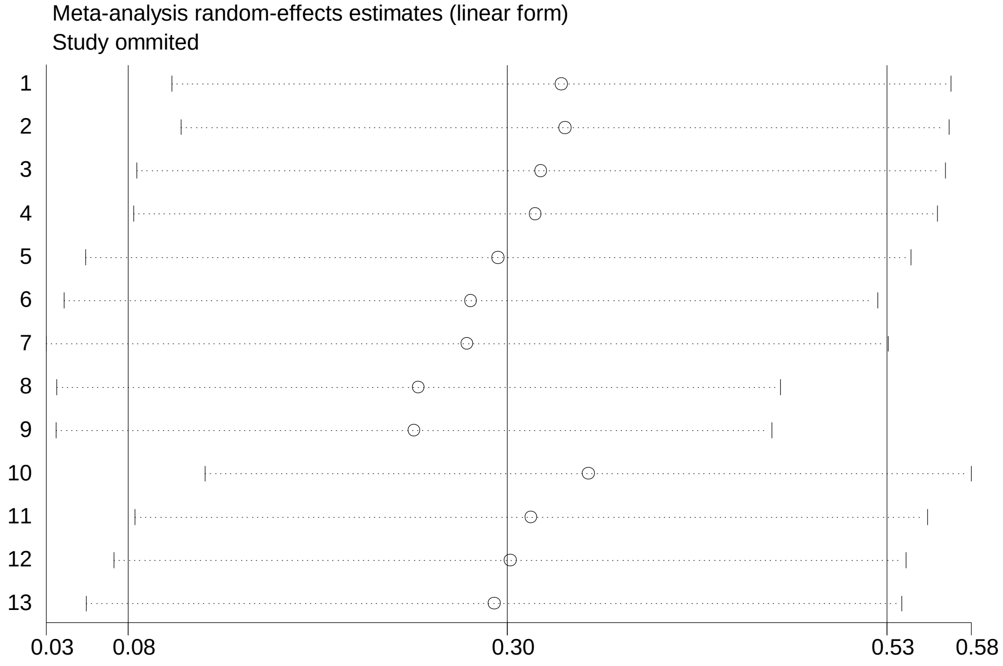

(b)

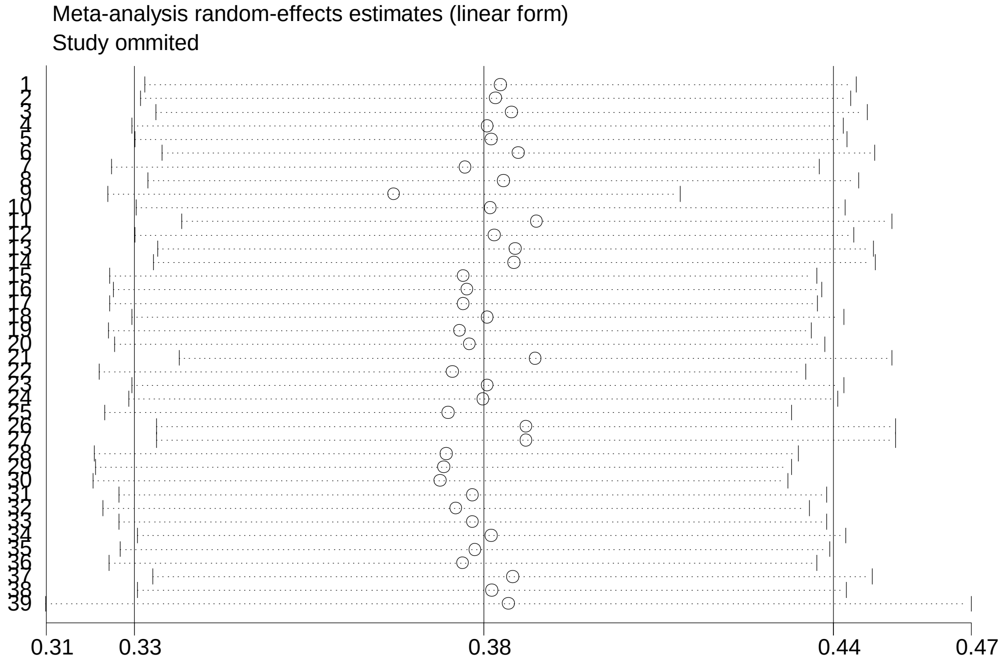
Meta-analysis random-effects estimates (linear form)
Study ommited
0.31
0.33
0.38
0.44
0.47

(c)

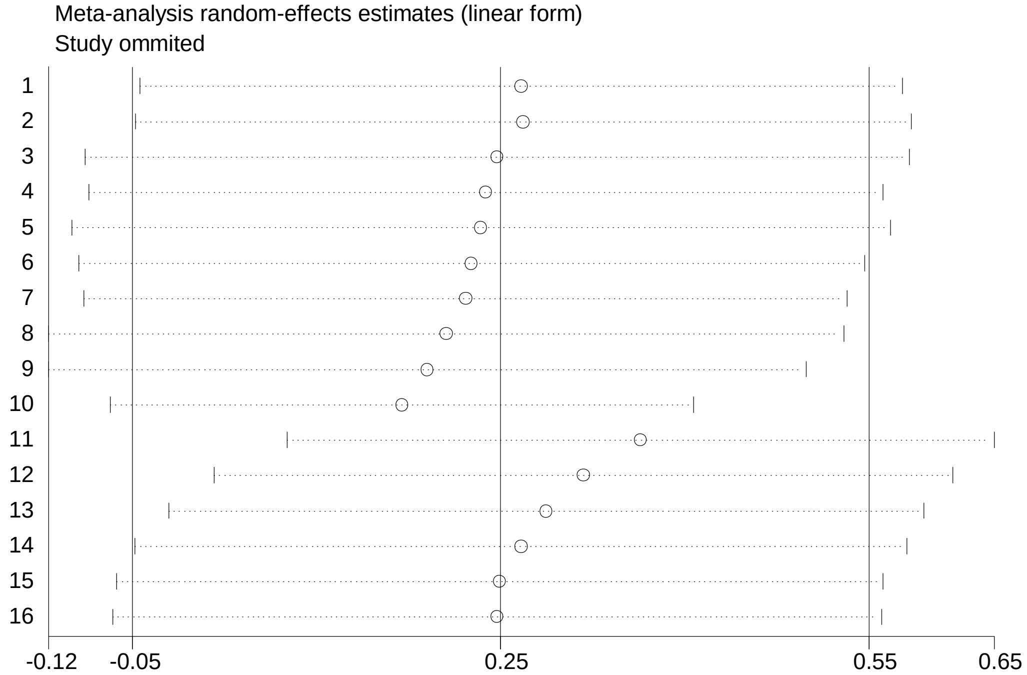
Meta-analysis random-effects estimates (linear form)
Study ommited
-0.12
-0.05
0.25
0.55
0.65

(d)
