# Supplementary material for: Prevalence and severity of anxiety and depression in Chinese patients with breast cancer: a systematic review and meta-analysis
Source: Front Psychiatry. 2023 Jun 28;14:1080413. doi: 10.3389/fpsyt.2023.1080413 (PMC10336240; doi:10.3389/fpsyt.2023.1080413)
Supplement: Supplementary file 5 [file Data_Sheet_5.pdf]

Supplementary Information 5

Results of publication bias (funnel plot and Egger's funnel plot)

(a) the prevalence of anxiety; (b)mean scores of anxiety; (c)the prevalence of depression; (d)mean scores of depression

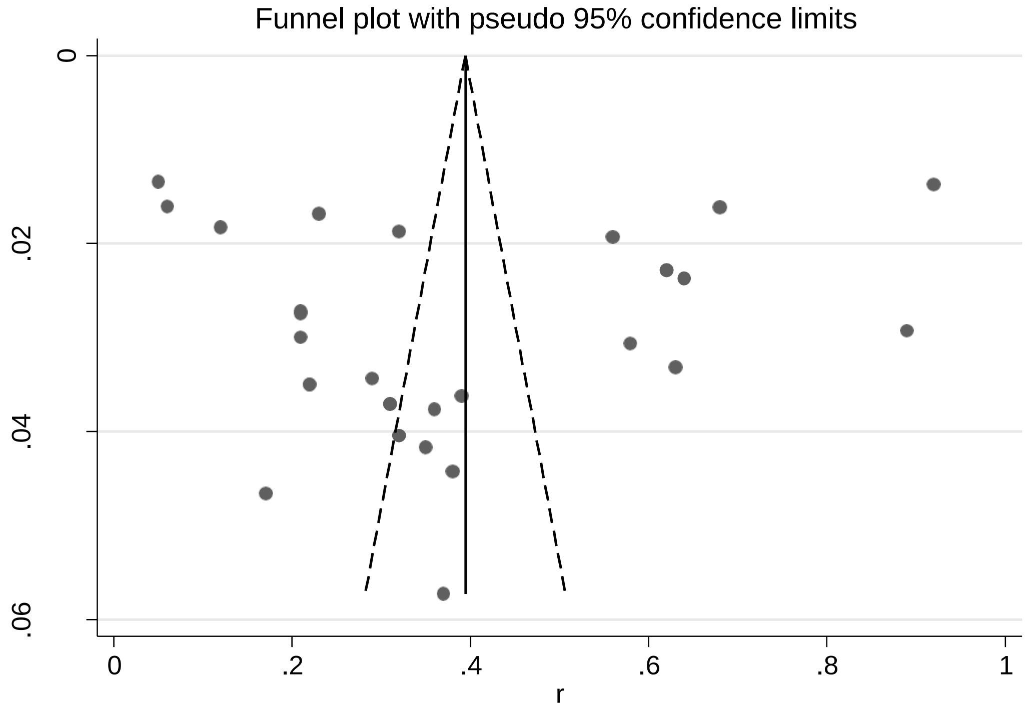

Egger's test

Std_Eff	Coef.	Std. Err.	t	P>t	[95% Conf. Interval]	
slope	.4220304	.1527518	2.76	0.011	.1074322	.7366286
bias	-1.265327	6.451211	-0.20	0.846	-14.55184	12.02119

(a)

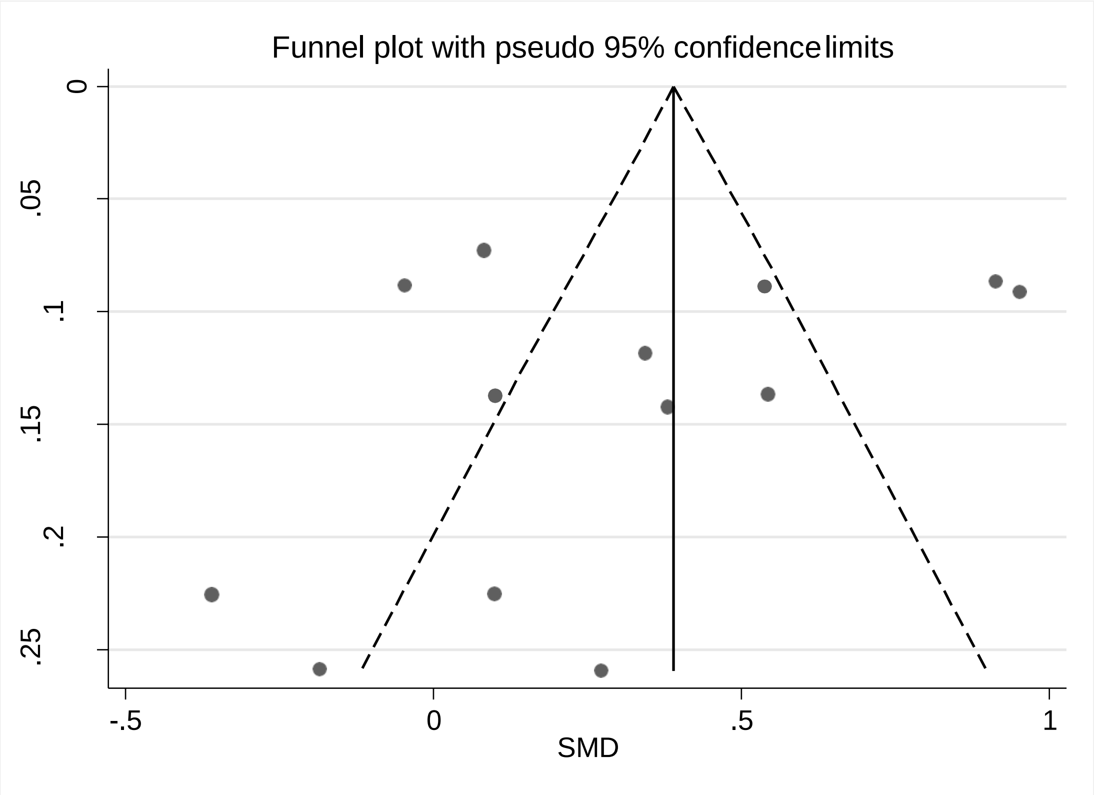

Egger’s test

Std_Eff	Coef.	Std. Err.	t	P>t	[95% Conf. Interval]	
slope	.6325693	.2985105	2.12	0.058	-.0244479	1.289586
bias	-2.273657	2.606131	-0.87	0.402	-8.009712	3.462398

(b)

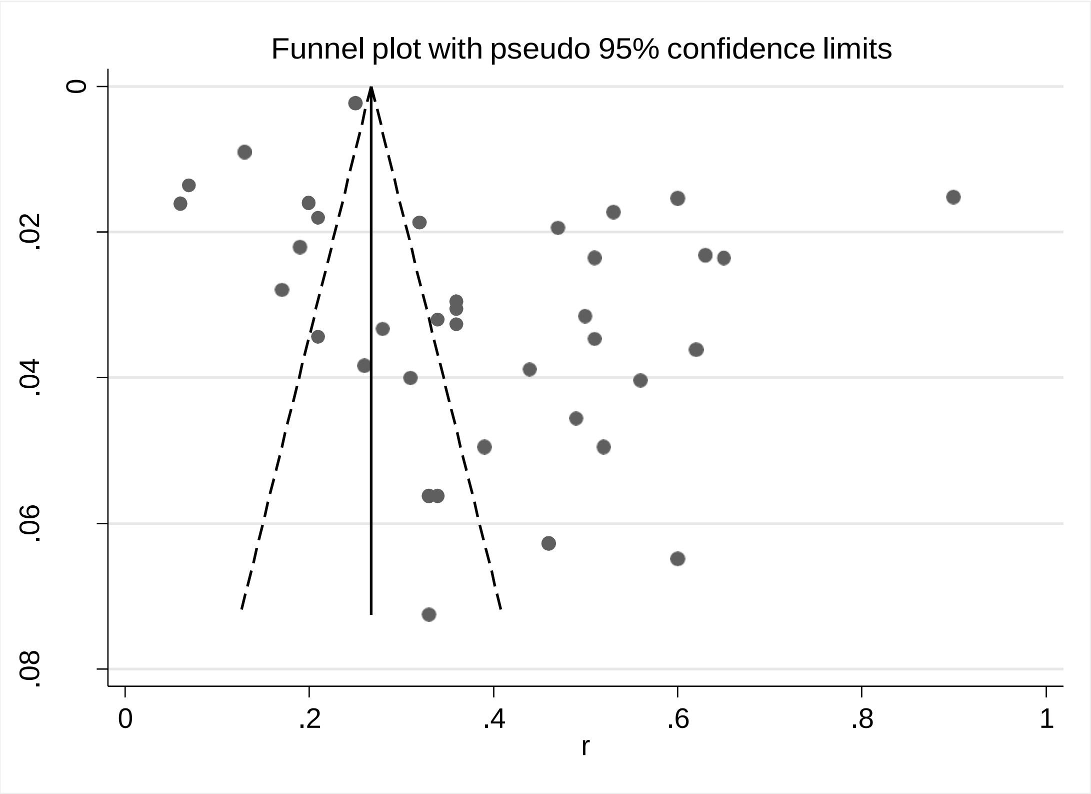

Std_Eff	Coef.	Std. Err.	t	P>t	[95% Conf. Interval]	
slope	.2312331	.0243198	9.51	0.000	.1819564	2805098
bias	5.081364	2.042241	2.49	0.017	.9433912	9.219337

(c)

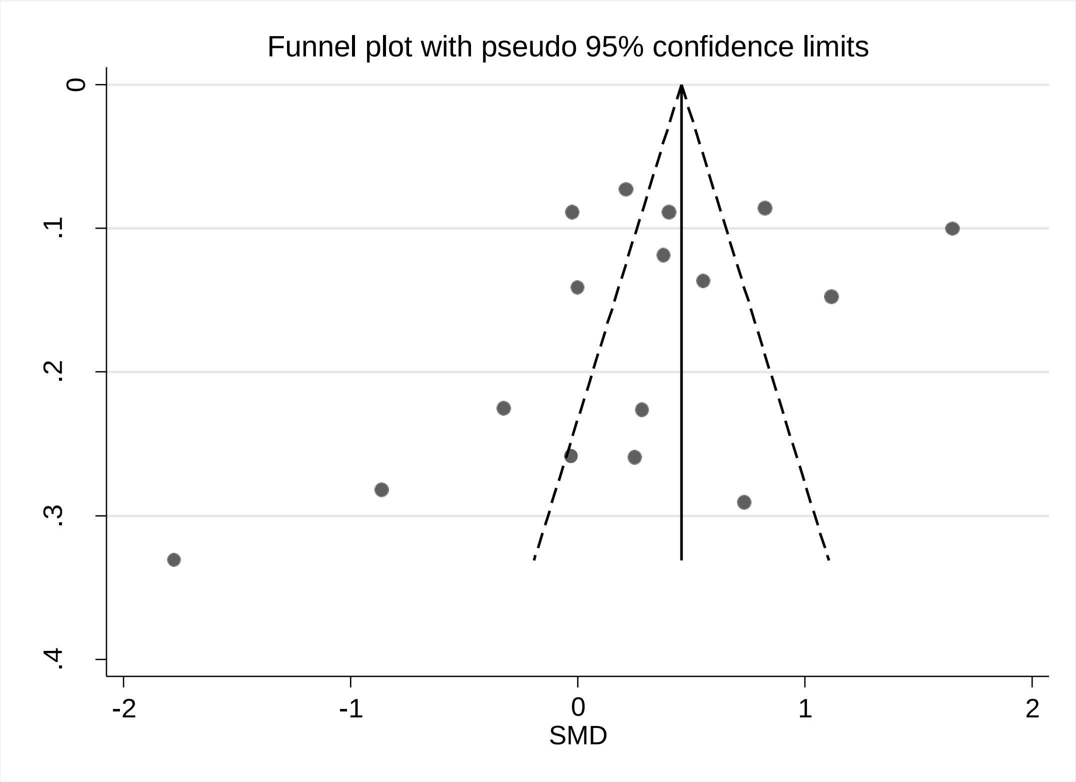

Std_Eff	Coef.	Std. Err.	t	P>t	[95% Conf. Interval]	
slope	.8058499	.3322653	2.43	0.029	.0932116	1.518488
bias	-3.05989	2.627803	-1.16	0.264	-8.695966	2.576187

(d)
